# Elevated body temperature exacerbates arrhythmia and seizure-like activity in a zebrafish model of Timothy syndrome

**DOI:** 10.1038/s42003-026-10657-3

**Published:** 2026-07-09

**Authors:** Svetlana A. Semenova, Amir M. Sams, Deepthi Nammi, Matthew Menold, Gennady Margolin, Jennifer L. Sinclair, Katelyn A. Robertson, Kerry Larkin, Sydney Kelly, Andy Golden, Benjamin Feldman, Harold A. Burgess

**Affiliations:** 1 Division of Developmental Biology, Eunice Kennedy Shriver National Institute of Child Health and Human Development, Bethesda, MD USA; 2 Bioinformatics and Scientific Programming Core, Eunice Kennedy Shriver National Institute of Child Health and Human Development, Bethesda, MD USA; 3 Laboratory of Biochemistry and Genetics, National Institute of Diabetes, Digestive, and Kidney Diseases, Bethesda, MD USA; 4 Zebrafish Core, Eunice Kennedy Shriver National Institute of Child Health and Human Development, Bethesda, MD USA; 5 Present Address: Department of Cell Biology, Yale School of Medicine, New Haven, CT USA

**Keywords:** Neurological disorders, Physiology, Genetics of the nervous system

## Abstract

Timothy syndrome (TS) is a multisystem disorder with autistic-like features, seizures and life-threatening arrhythmias. Most TS cases are caused by a de novo missense mutation in the *CACNA1C* gene that encodes the pore-forming subunit of the voltage-gated L-type calcium channel Ca_V_1.2. Patients show highly variable clinical presentations and survival, which may be impacted by acute episodes of hypoglycemia and respiratory dysfunction. In addition, fever, known to activate Ca_V_1.2 channels, may exacerbate TS, yet its impact is poorly understood. We generated a zebrafish model carrying the homologous *cacna1c* mutation. These fish exhibited arrhythmias, increased susceptibility to pentylenetetrazole-induced seizures, microcephaly, cerebellar hypotrophy and abnormal GABAergic neuron development. Transcriptomic analysis revealed dysregulated expression of neuropeptides including *bdnf* and *vgf*. Notably, elevated water temperature elicited arrhythmia and seizure-like behavior even in overtly normal heterozygotes, implicating fever as a modifiable risk. This model provides a platform for dissecting TS pathophysiology and identifying strategies to mitigate fever-related risk.

## Abstract (web summary)

Zebrafish with a Timothy syndrome mutation in *cacna1c* recapitulated arrhythmias and seizure-susceptibility reported in patients. Heat exacerbated these phenotypes suggesting that fever may be a modifiable health risk.

## Introduction

Timothy syndrome (TS) is a rare autosomal dominant disorder caused by a de novo gain of function mutation in *CACNA1C*, the gene encoding the pore-forming α-1 subunit of the L-type voltage-gated calcium channel (LTCC) Ca_V_1.2. Major symptoms of TS include cardiac arrhythmia with QT interval prolongation, cutaneous syndactyly and autistic behaviors (reviewed in ref. ^1^). Most patients have a G406R missense mutation in either of two mutually exclusive, alternatively spliced isoforms of exon 8; mutations in exon 8A cause Timothy Syndrome 1 (TS1), whereas mutations in exon 8 cause Timothy Syndrome 2 (TS2). Each exon 8 variant encodes 34 amino acids with partially similar amino acid sequences, and channels with mutations in either exon 8 or 8A show increased inward calcium current due to a reduction in voltage-dependent inactivation^2,3^. Clinical features show a high degree of variability, that may reflect differences in genetic background or mosaicism, especially in light of complex tissue-specific patterns of *CACNA1C* alternative splicing^4–6^. However, acute challenges such as hypoglycemia, anesthesia and respiratory distress may also contribute to variable outcomes. Indeed, a recent TS2 mouse model demonstrated abnormal activity in *CACNA1C* expressing hypothalamic glucose-sensing neurons that may relate to patient hypoglycemia^7^, emphasizing the value of models for dissecting pathophysiology associated with potentially preventable triggers.

Notably, fever has an established role in aggravating other long QT syndromes^8^ including in Brugada syndrome, where fever-induced ventricular fibrillation carries a high risk of sudden death^9^. Intriguingly, the Ca_V_1.2 channel has an unusual property of spontaneously opening at temperatures above 37 °C, leading to cellular depolarization^10^. This may be intensified in TS Ca_V_1.2 channels, which show delayed inactivation. Moreover, because TS patients are prone to infections, elevated L-type calcium channel activity during fever may lead to tachyarrhythmia, which is a major cause of mortality; in fact infection was reported as an associated factor for sudden death in a TS case report^11^. Together, this highlights fever as a potentially mitigatable condition for patients. However, the impact of fever in TS is uncertain and difficult to study without a suitable model.

In zebrafish, body temperature can be precisely controlled by adjusting the temperature of the surrounding water. Moreover, animals are raised at 28 °C, well below the spontaneous opening threshold for Ca_V_1.2 channels. A zebrafish *cacna1c* loss of function allele caused developmental defects in ventricular cardiomyocytes and mandible; however, gain of function phenotypes relevant to Timothy syndrome have not been examined^12,13^. We therefore generated a zebrafish knock-in model with a mutation homologous to the TS2 exon 8 G406R mutation, compared phenotypes to clinical manifestations and tested the effects of elevated water temperature on behavior and physiology.

Homozygous mutants showed a range of phenotypes reminiscent of symptoms in patients. At embryonic stages, both cardiac chambers showed reduced contraction frequency, reminiscent of prenatal and early postnatal bradycardia reported in patients^5^. Several days later, contraction frequency normalized but mutants demonstrated an intermittent 2:1 atrioventricular block, recapitulating a characteristic patient arrhythmia. Epilepsy is present in a high proportion of patients, and accordingly, homozygous mutants showed heightened sensitivity to pentylenetetrazol, a seizure-inducing agent. We used brain-wide morphometry to systematically assess changes in brain structure and composition. Mutants showed mild microcephaly and a disproportionately severe reduction in cerebellar tissue adjacent to the midbrain-hindbrain boundary (MHB). Differentiated neuron markers were inappropriately present at the midline in mutants, and inhibitory neuron markers were altered in a region-specific manner. Importantly, heterozygous larvae did not manifest cardiac abnormalities or seizure-like behavior under normal conditions, but when tested at elevated temperature produced seizures and arrhythmias. These findings establish a new model for studying the pathophysiology of TS2 and support the possibility that fever is a preventable risk in TS patients.

## Methods

### Experimental design

All experiments were performed with at least two independent cohorts of larvae up to 7 dpf, before sex differentiation. Numbers for brain imaging and behavioral experiments were based on previous work^14,15^. Larvae used in seizure experiments, GCaMP imaging and temperature-induced tachycardia experiments were derived from a heterozygous incross and genotyped after the experiment, so that the investigator was blind to the genotype. Due to technical constraints, brain morphometry and analysis of heart rate after splice switching were performed on pre-genotyped larvae and not performed blind. All data are included (no data points were excluded). We have complied with all relevant ethical regulations for animal use.

### Husbandry

Embryos and larvae were raised in a 14:10 light:dark cycle at 28 °C (except as otherwise noted) in E3 medium supplemented with 1.5 mM HEPES pH 7.3 for buffering. Transgenic lines used in this study were *TgBAC(gad1b:lox-RFP-lox-GFP)nns26* (*gad1b:RFP*)^16^ and *Tg(Cau.Tuba1a:MCardinal)y516* (*tuba:mCardinal*)^14^.

### CRISPR-Cas9 mutagenesis and fish line maintenance

A four-nucleotide substitute that leads to a G419R amino acid substitution in the zebrafish CACNA1C protein was introduced into the genome of ABC strain fish by CRISPR-Cas9 knockin (In Vivo Biosystems, Eugene, OR). This allele, NM_131900.1(cacna1c):c.1252_1255delAGCGinsTCTA, designated *y680*, was originally made in an ABC background, and maintained by repeatedly crossing heterozygous carriers to TL wild-type stock (Zebrafish International Resource Center, Eugene, OR). For genotyping, we performed PCR with DNA from tail fins and primers 5-GTTTCCGCTCACTGTCTTCC and 5-ACACGCATTTAGCACACAATGCTTATC, then digested PCR products with XbaI for 2 h at 37 °C and separated on a 1% agarose (biotechnology grade, VWR) + 2% MetaPhor agarose (Lonza) gel. The wild-type 202-bp PCR product was intact, while mutant PCR products were cleaved to a 170-bp fragment.

### Brain morphometry

For brain morphometry experiments *cacna1c*^y680+/−^*; gad1b:RFP; tuba:mCardinal* fish were backcrossed to *cacna1c*^y680+/−^, and fin clips from offspring were collected at 3 dpf for genotyping. Brains of live larvae were imaged at 6 dpf on a Nikon A1 confocal microscope with a 20× water immersion objective, voxel size 1 × 1 × 2 μm. Z intensity correction was used to compensate for weaker signals from the ventral-most parts of the brain. After rotation to orient the main axis of the brain horizontally and channel separation in ImageJ, confocal stacks were aligned to the *gad1b:RFP* and *tuba:mCardinal* patterns from Zebrafish Brain Browser (ZBB) using multi-channel registration in ANTS^17^. After registration, we calculated the log Jacobian determinant of the transformation matrix to generate a map for each fish, showing the extent to which individual voxels were contracted or dilated to match the reference brain (LJD channel). Next, we used Cobraz to identify clusters of voxels that differed in wild-type and mutant larvae in the *gad1b:RFP*, *tuba:mCardinal* or LJD channels, by using permutation analysis to rigorously establish significance thresholds for each channel^14^. We only reported changes that were consistent between experiments performed in two independent cohorts of larvae. In addition, we symmetrized maps of significant voxels by taking the greater p-value at each bilaterally symmetric position. To measure total brain volume, neuroanatomical annotations in the ZBB reference brain were inverse-transformed onto the original brain scans. Similarly, to measure changes in cerebellar or midline volume in mutants, we created masks covering the affected region in the first experiment and, in the second cohort of fish, inverse-transformed it back onto the unprocessed brain scans, allowing us to count voxels within the mask in wild-type and mutant brains and thereby determine the volume of the affected area. Imaging and brain morphometry procedures were as previously described^18^.

### Morpholino injections

To assess morpholino efficacy, fertilized wild-type embryos were injected with standard control morpholino (Gene Tools) or 1 ng or 3 ng of cacna1c-i7e8 morpholino (5-CATCCTGCATCTGAGACGAGGAGGG). At 2 and 5 dpf, embryos were dissected caudal to the yolk, and anterior tissue (including head and heart) was collected for mRNA preparation using the Maxwell RSC simplyRNA kit. We used SuperScript™ III One-Step RT-PCR System with Platinum™ Taq High Fidelity DNA Polymerase with primers 5-TTTGCCATGCTGACTGTGTT and 5-GTGATCCAGTCCAGGTAGCC producing a 275 bp band for either exon 8 or exon 8A containing isoforms. We then digested the product with EcoRI: exon 8 has no EcoRI target, whereas exon 8A is cleaved into 195 and 80 bp fragments. A dose-dependent shift in exon 8 splicing was apparent at 2 dpf (Supplementary Fig. 4B). For testing cardiac contraction frequency, wild-type and mutant cousin embryos (i.e., embryos that were derived from parents that were wild-type and mutant siblings, respectively) were injected with 3 ng control or i7e8 morpholino, then tested at 2 dpf.

### Survival monitoring

Fish were genotyped as larvae, then raised with siblings of the same genotype in groups of 4–6 per 1.8 L tank and counted daily. For the survival monitoring in large groups, fish were genotyped at the larval stage and placed into 1.8 L tanks in groups of 20–29 (2 groups of 10 heterozygotes, 5 wild-type, 5 mutants, and 1 group of 12 heterozygotes, 7 mutants, 10 wild-type) at the age of 6 dpf. Fish were counted and genotyped after they reached the adult stage. Photographs of the adult fish taken from above were processed in ImageJ to measure body length (from the tip of the mouth to the base of the caudal fin).

### Expression analysis

To compare expression of *cacna1c* to markers of stages of neuronal maturation, we downloaded the DanioCell single cell sequencing dataset from NCBI GEO (accession GSE223922^19^,) and extracted cells annotated as ‘neural’. For each gene of interest, we normalized expression to total UMIs per cell, then calculated mean expression across cells of a given age range. Expression values for each gene were normalized to maximal expression across all ages then smoothed for plotting.

### Heart rate measurements

Larval fish were anesthetized with tricaine to prevent spontaneous movements and placed under a microscope connected to a Chronos 1.4 high-speed camera, allowing heart videos to be recorded at 100 frames per second. Tricaine anesthesia was reported to reduce the heart rate in larval zebrafish^20^ however, spontaneous movements of the fish can render video recordings unusable for heart rate assessment and therefore applying sufficient tricaine to immobilize the larvae was necessary. We used ImageJ to calculate mean pixel intensity over time in ROIs that covered the atrial/ventricular wall^21^. Time series data was processed in Python to compute spectrograms (which represent the power present at different frequencies in the Fourier transform of mean image intensity from each cardiac chamber over time) using the *spectrogram* function in the scipy.signal library^22^. We used the *find_peaks* function in scipy.signal to determine mean ventricular and atrial contraction rates for experiments at 28 °C but found this was not sufficiently accurate at high contraction frequencies for larvae recorded at 37 °C, so instead, we extracted the dominant contraction frequency using the Fourier transform in scipy.signal *spectrogram*. To measure contraction frequency at 37 °C we placed larvae in the center of a glass Petri dish wrapped with a foil Kapton flexible heating element (Omegalux) powered by a TC200 Temperature Controller (Thorlabs), with a 10 kΩ thermistor to measure temperature. We verified the temperature in the center of the dish using an I3 infrared thermal imager (FLIR). Exposure to 37 °C is widely used to activate transgenic heat-shock promoters in zebrafish larvae and does not cause negative effects^23^.

### RNA analysis

To prepare samples for transcriptomic analysis, embryos (2 dpf) and larvae (6 dpf) were cut in the middle of the yolk extension under terminal anesthesia. Tail tissue was used for DNA isolation and genotyping, and the anterior part of the body (mostly head) stored in RNAlater (ThermoFisher) at –20 °C. Three heads from larvae of the same genotype were pooled, and RNA was isolated in a Maxwell 16 instrument with a Maxwell 16 LEV Simply RNA Tissue kit (Promega). Capillary electrophoresis in an Agilent Bioanalyzer was used for RNA quality control. cDNA libraries were prepared and sequenced at the Molecular Genomics Core, NICHD. We used FastQC v.0.12.1 to check FASTQ file quality and trimmed sequences with Cutadapt v.4.4 before aligning to zebrafish genome version GRCz11 using STAR v2.7. We then used featureCounts from the Subread package v2.0.3 to generate gene counts from BAM files. Genetic background associated polymorphisms that control gene expression levels frequently cause differences in expression of genes on the same chromosome as an experimental mutation that are linked to the mutation rather than being effects of the mutation itself^24^. We expected this phenomenon to be exacerbated in TS2 mutant larvae because the *y680* mutation was made on an ABC wild-type background, and subsequently crossed to a TL background. Indeed, differentially expressed genes (DEGs) were disproportionately located on chromosome 4, the same chromosome as *cacna1c* (70.6%, Supplementary Fig. 3A). Therefore, for annotation of DEGs and Gene Ontology pathway analysis, we excluded genes located on chromosome 4. We used DESeq2 v1.33.0 with apeglm shrinkage to measure differential expression. We removed genes with less than 100 mean counts, and applied the Benjamini–Hochberg false discovery rate (FDR) correction to DESeq2 *p*-values and considered genes with an FDR of <0.1 as significant. Gene pattern analysis was performed with DEGreport version 1.34.0, utilizing the ConsensusClusterPlus algorithm to cluster genes based on similar expression profiles, using differentially expressed genes between wild-type and mutant as input (FDR < 0.1, but not filtering for base mean expression)^25^. For gene ontology analysis, lists of ENSEMBL identifiers for upregulated and downregulated genes were processed with the ShinyGO web tool (https://bioinformatics.sdstate.edu/go/^26^;) with FDR cutoff set to 0.05, number of pathways to show set to 500, and “remove redundancy” option flagged. Daniocell (daniocell.nichd.nih.gov) and zfin.org databases were used to annotate the functions and tissue specificity of expression of the differentially regulated genes^19,27^. To compare splicing in wild-type and mutant larvae, we used *featureCounts* from the subread package to measure splice junctions, then normalized the junction counts for each sample using scaling factors generated by deepTools. Independent RNA samples prepared in the same manner were used for quantitative RT-PCR to characterize the usage of mutually exclusive *cacna1c* exons 8 and 8A at 2, 4, and 6 dpf. cDNA synthesis and qPCR were performed at BioInnovatise (Rockville, MD). Primers are listed in Supplementary Table 1, including for the reference gene *eef1a1l1* (*EF1α*) for which we used previously described primers^28^.

### Brain activity measurements

To measure neural activity, we first crossed heterozygous fish with the TS2 mutation to *Tg(elavl3:H2B-GCaMP6s)jf5*^29^, then backcrossed to heterozygous fish. We embedded larvae in agarose and used epifluorescent imaging to record neural activity at 0.5 Hz for 5 min. We then extracted DNA for post-hoc genotyping. To correct for drift over the recording period, we used ImageJ to perform a rigid registration on slices in the virtual stack. We then used ANTs to co-register recordings. We applied a 2 sigma Gaussian filter from *scipy.ndimage* to correct for subpixel drift and noise in each image. To measure fluorescence signals, we first calculated the mean signal intensity within a mask that encompassed cellular brain regions in images within each time-series. We then detrended the time-series signal to account for GCaMP photobleaching and differences in baseline between larvae, then measured the area under the resulting curve in each recording.

### Hybridization chain reaction (HCR)

Fluorescent in situ hybridization to detect gene expression was performed according to the manufacturer’s protocol (Molecular Instruments) with the proteinase K treatment step omitted and the samples partially dissected (tail cut off just rostrally to the yolk extension under terminal anesthesia prior to fixation). Oligonucleotide probes for *cacna1c, vgf, sox2* and *en1b* were used with the B3 amplifier, and probes for *elavl3* were used with B1 or B2 amplifier. Probes for *elavl3* with the B2 amplifier were from Molecular Instruments, and other probes were designed in-house^30^. Samples were stored in PBS pH 7.4 at 4 °C prior to imaging. Probe sequences are in Supplementary Data 3.

### Behavioral analysis

To measure seizure-like activity, larvae derived from a heterozygous incross were transferred to individual wells of a 36-well testing arena filled with E3 embryo medium with 1% DMSO (vehicle control) or freshly prepared 1 mM PTZ in E3 with 1% DMSO. Wells had dimensions 7 × 7 mm. After 30 min exposure to PTZ, locomotor activity was recorded for 15 min; then the light was extinguished and activity recorded for another 15 min. Larvae were tracked in real-time at 10 Hz using DAQtimer then post-hoc genotyped^31^. After recordings were complete, we calculated the total distance moved for each larva in 1 s windows, then calculated a large-movement distance threshold that was exceeded in only 0.1% of windows in wild-type vehicle-treated larvae – thus over a 15 min recording period, we would expect less than one such movement in untreated wild-type larvae (Supplementary Fig. S5A, B). We then recorded the total number of instances in which this threshold was exceeded in 1 s windows for each larva in illuminated and dark conditions. To assess startle behavior and prepulse inhibition, the responses of the larvae to vibrational stimuli were filmed at 1000 frames per second with a high-speed camera and analyzed with Flote software^15^. We performed two replications of this experiment, and for each measure we assessed significance using ANOVA to calculate the main effect of genotype, controlling for the experiment and controlling the false discovery rate with Benjamini–Hochberg correction. To assess activity for larvae at 37 °C, we used the same temperature controller as for cardiac contraction experiments, with larvae in a 4-well grid inside the chamber, with each well having the same dimensions as above.

### Statistics and reproducibility

Data were analyzed using Python 3.7, R and JASP). Estimation plots were generated using the *dabest* package and represent means and effect size (Hedges’s g)^32^. All *t* tests are two-tailed and independent sample Welch tests. For data with outliers or non-linear Q-Q plots we used Mann–Whitney U tests (MWU). In box and whisker plots, box shows the 25–75 percentile range, and whiskers show 1.5× the interquartile range. The random allocation of larvae to the experimental groups (wild-type, heterozygous, or knockout) was driven by Mendelian inheritance, with larvae tested blind unless otherwise noted and genotyped after the experiment. Replicate experiments were performed with cohorts of larvae derived from independent crosses.

### Reporting summary

Further information on research design is available in the Nature Portfolio Reporting Summary linked to this article.

## Results

### Zebrafish with the Timothy syndrome 2 mutation are viable with increased neuronal activity

**Fig. 1:**
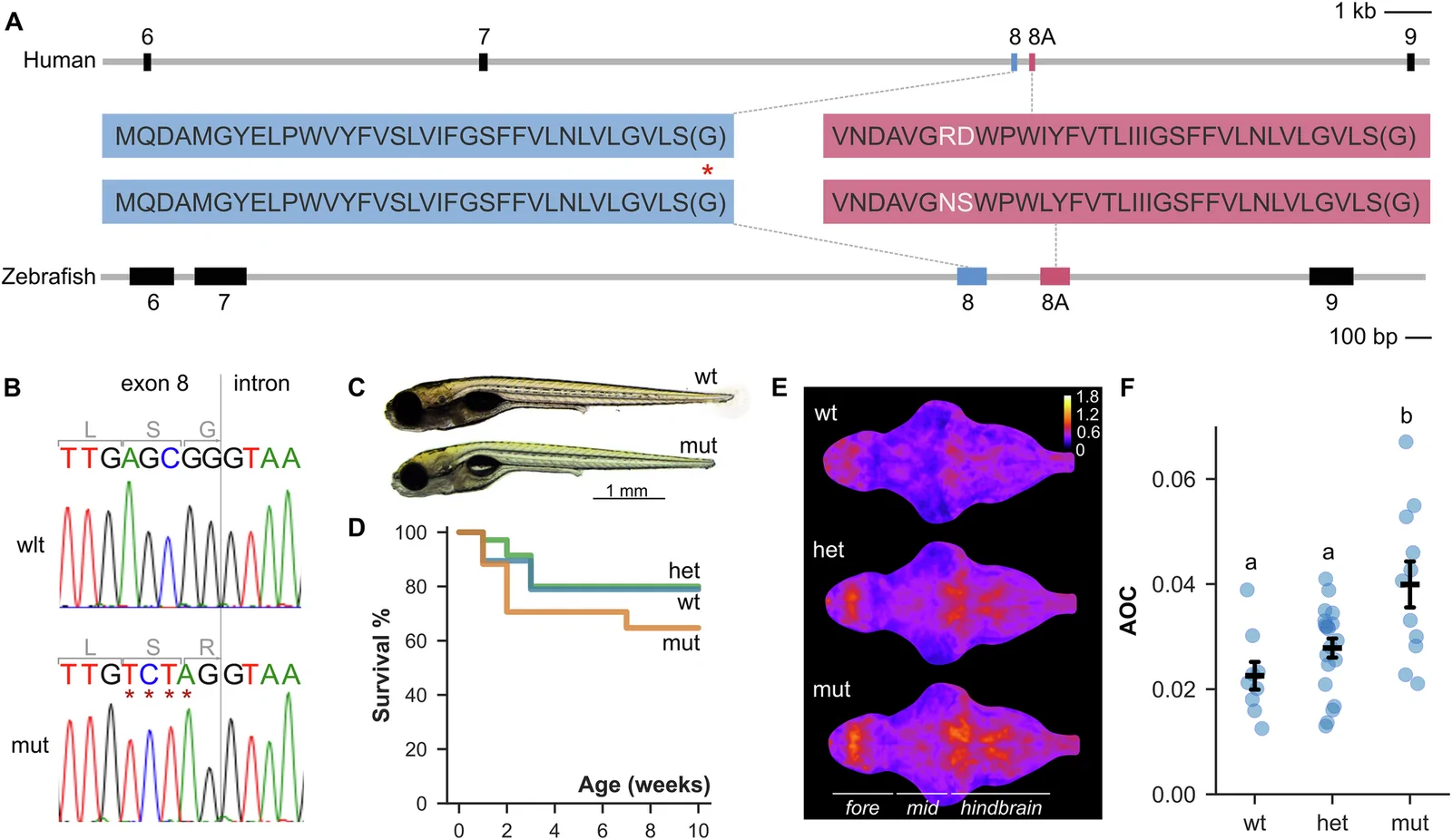
A zebrafish knock-in model for Timothy syndrome 2. **A** Mutually exclusive exons 8 and 8A are conserved in zebrafish *cacna1c* and human *CACNA1C* genes. The symbol for the glycine residue mutated in TS (indicated by red asterisk) is shown in parentheses because the third base of the codon for this residue is contributed by exon 9. **B** Sequence from wild-type (top) and mutant (bottom) larvae. Red asterisks indicate the nucleotides edited (mutation that converts the final codon in exon 8 from encoding Gly to Arg and a silent mutation in the penultimate codon for Ser in exon 8, AGC to TCT, introduced to facilitate genotyping). **C** Wild-type and homozygous mutant larvae at 6 dpf. **D** Kaplan–Meier survival plot for wild-type, heterozygous and mutant larvae during development to adulthood (*N* = 19, 35, 17 larvae respectively). **E** Mean GCaMP dF/F in 6 dpf wild-type, heterozygous and mutant larval brains (*N* = 9, 20, 11 animals respectively). Forebrain, midbrain, hindbrain indicated. Color bar shows dF/F. **F** Area under the curve (AOC) for GCaMP signal within cellular brain area for samples in (**E**). Mean and standard error of mean indicated for each genotype. ANOVA F_2,37_ = 8.0, *p* = 0.001. Letters indicate groups that were significantly different in post-hoc Tukey tests.

Human and zebrafish Cacna1c protein sequences are highly conserved, with 78.9% identity. We examined genomic sequence in zebrafish to assess the feasibility of generating a zebrafish mutation that precisely recapitulated patient mutations in Timothy syndrome. The G406R mutation in Timothy syndrome is due to a missense mutation that can occur at the end of either of the two mutually exclusive exon 8 variants, exon 8 or exon 8A. Patient mutations that affect either exon 8 or 8A have overlapping clinical manifestations, including autism, seizures and cardiac arrhythmia. However, mutations in exon 8 lead to worse outcomes than 8A mutations, and present other differences such as a lack of syndactyly^3^. These phenotypic variations may be because exon 8 is around 4-fold more abundant in brain and heart tissue, or because exons 8 and 8A show distinct temporal patterns of expression during neural development. Similarly, the zebrafish homolog of *cacna1c* comprises alternate exons 8 and 8A, which encode amino acid sequences that are 100% and 94% identical to human exons 8 and 8A, respectively (Fig. 1A). Quantitative PCR at 2, 4 and 6 dpf did not detect an age-related change in exon 8 usage vs exon 8A (F_2,10_ = 2.89, *p* = 0.11), and exon 8 was at least 13-fold more abundant than exon 8A at all ages (Supplementary Fig. S1A).

We generated a G419R missense mutation (corresponding to human G406R) at the end of the more highly expressed exon 8, thereby creating allele *cacna1c*^*y680*^ as a potential model for Timothy syndrome 2 (TS2 mutant, Fig. 1B). Homozygous mutant (herein ‘mutant’) and heterozygous TS2 larvae were overtly similar to their wild-type siblings in body size and overall appearance (Fig. 1C). Early mortality is common in TS patients. Only 23% of mutants from a TS2 heterozygous incross survived to adulthood when raised in mixed genotype pools, significantly less than the survival rates of 69% and 90% for heterozygous and wild-type fish, respectively (ANOVA main effect of genotype, F_2,6_ = 25.037, *p* = 0.001; Supplementary Fig. S1B). However, the mortality of mutants raised in same-genotype pools was only 18% greater than that of wild-type siblings; one possibility is that diminished survival in mutants raised in mixed-genotype groups reflects a reduced ability to compete with siblings (Fig. 1D). Surviving mutants were sexually dimorphic and fertile, although males were smaller than wild-type siblings (ANOVA, interaction of genotype and sex, F_2,50_ = 3.5, *p* = 0.037 ; Supplementary Fig. S1C). Because the G419R mutation resulted from nucleotide changes at the 3’ end of exon 8, we questioned whether the relatively normal growth and survival observed in mutants might be due to a splicing change that minimized the mutation’s effects by increasing expression of isoforms incorporating the wild-type exon 8A. However, we found no significant changes in the frequency of splicing into or out of either exon 8 or 8A in mutants, making it unlikely that splice changes mask the mutant phenotype (Supplementary Fig. S2). Thus, unlike in mammals where TS mutations are dominant and lead to high mortality, heterozygous TS2 zebrafish are overtly normal and even homozygotes are viable and fertile^7^.

We anticipated that the TS2 mutation would cause elevated activity in *cacna1c-*expressing neurons due to sustained depolarization. To test this, we introduced the mutation into fish with pan-neuronal GCaMP expression and measured fluorescence intensity over a 5 min period for each larva. Mutant larvae showed greater changes in fluorescence compared to wild-types, confirming that the TS2 mutation leads to increased neuronal activity (Fig. 1E, F, Supplementary Video 1). Activity was elevated in all brain subdivisions, with the largest increase seen in the prepontine division of the hindbrain (Supplementary Fig. S1D).

### TS2 mutants show increased neuropeptide expression

**Fig. 2:**
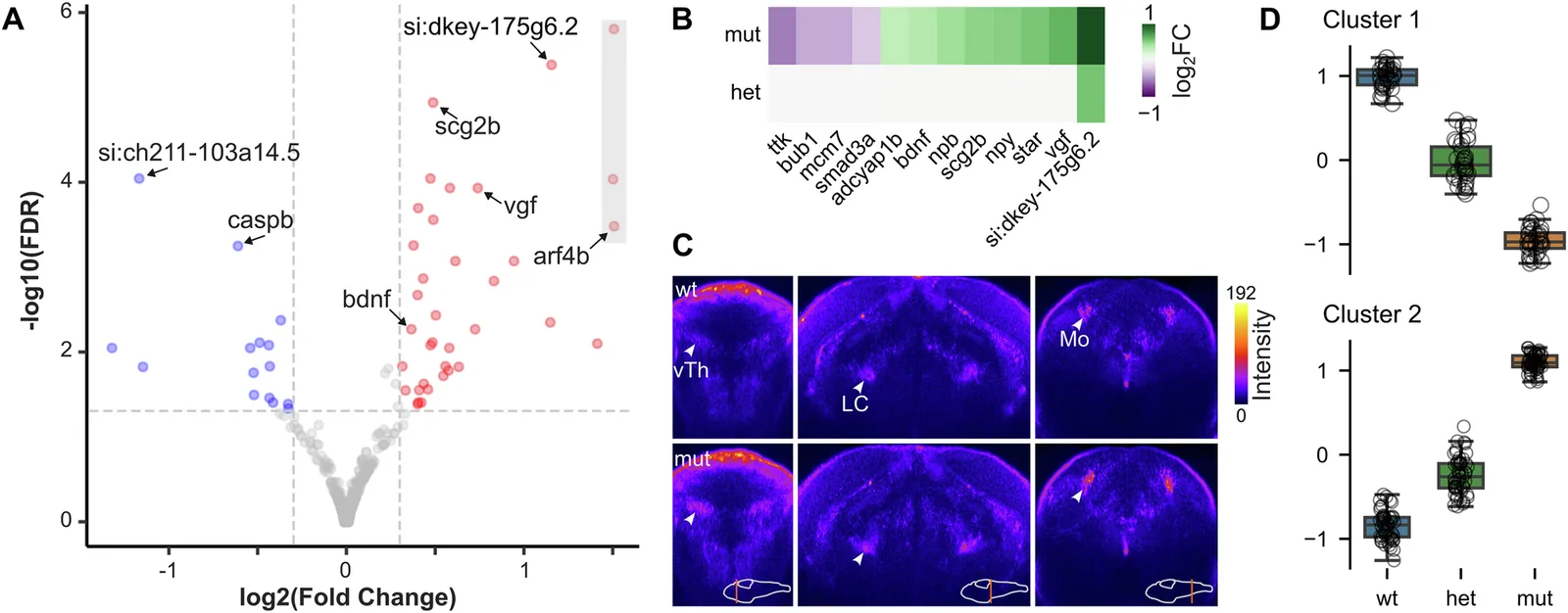
Changes in neuropeptide gene expression. **A** Genes differentially expressed between wild-type and mutant 6 dpf larvae using RNA-seq from anterior tissue (includes head and heart). Values for points in gray rectangle truncated for display. **B** Log2 fold-change in expression in mutant larvae compared to wildtype larvae for indicated genes. **C** Pseudocolored (see color scale bar) coronal sections through the diencephalon, pons and medulla after HCR fluorescent in situ hybridization for *vgf* in wild-type (top panels) and mutant (bottom) 6 dpf larvae. Images are the mean of multiple co-registered larval brain scans (*N* = 9, 8 larvae for wild-type and mutant, respectively). Schematic shows the position of each section. vTh ventral thalamus, LC locus coeruleus, Mo medulla oblongata. **D** Normalized expression of genes present in the two largest clusters from the *ConsensusClustersPlus* clustering algorithm, each comprising genes with correlated levels of expression in wild-type, heterozygous and mutant larvae. Input genes were defined based on differential expression between wild-type and mutant larvae, revealing an intermediate gene expression phenotype in heterozygotes.

Next we assessed changes in gene expression, using RNA-seq to compare wild-type, heterozygous and homozygous TS2 larvae at 2 and 6 dpf. We prepared total mRNA after removing the entire trunk caudal to the swim bladder to reduce the contribution of skeletal muscle genes and enrich for genes expressed in the brain and heart. At 2 dpf, 306 genes were differentially expressed in homozygous mutants compared to wild-types (149 downregulated, 157 upregulated; Supplementary Fig. S3A, B; Supplementary Data 1). Pathway analysis revealed significant upregulation of genes involved in muscle development and cardiac muscle contraction (Supplementary Fig. S3C, D), and downregulation of genes involved in mRNA splicing (Supplementary Fig. S3E). At 6 dpf, 46 genes were significantly upregulated and 25 genes downregulated in mutants (Fig. 2A; Supplementary Data 2). Downregulated genes were involved in cell cycle (*bub1, mcm7, ttk, smad3a*), whereas upregulated genes were implicated in neuropeptide hormone signaling or expressed in the anterior hypothalamus of larval zebrafish where many peptidergic neurons are located (*bdnf, vgf, si:dkey-175g6.2, npb, npy, adcyap1b* and *star*; Fig. 2B)^33^. *vgf* and *si:dkey-175g6.2* are paralogs of mammalian VGF, and are known to be positively regulated by *bdnf*. Because VGF has been implicated in neural development in mice, we examined *vgf* expression in mutants using fluorescent in situ hybridization^34,35^. *vgf* was expressed in discrete clusters in the pallium, subpallium, ventral thalamus, raphe, locus coeruleus and medulla oblongata, and mutants showed higher expression than wild-type siblings in each of these areas (Fig. 2C).

We also compared heterozygous mutants to wild-type siblings at 6 dpf. Only 10 genes were differentially expressed, including upregulation of the *vgf* paralog *si:dkey-175g6.2* (Supplementary Fig. S3F). However, to examine whether subtle changes were present in heterozygous larvae, we applied the ConsensusClusterPlus algorithm using the subset of genes differentially expressed between 6 dpf wild-type and homozygous mutants to perform unsupervised clustering of genes into groups based on correlated expression in wild-type, heterozygous and mutant larvae. Most (95%) genes were assigned to two large clusters corresponding to genes that were downregulated and upregulated in mutants, respectively. Interestingly, mean expression levels in heterozygous animals were midway between wild-type and mutant levels in each cluster (Fig. 2D). Thus, the changes in gene expression in overtly normal heterozygous larvae are similar to, but less severe than in homozygous mutants.

### Mutants exhibit embryonic bradycardia and intermittent cardiac arrhythmia

**Fig. 3:**
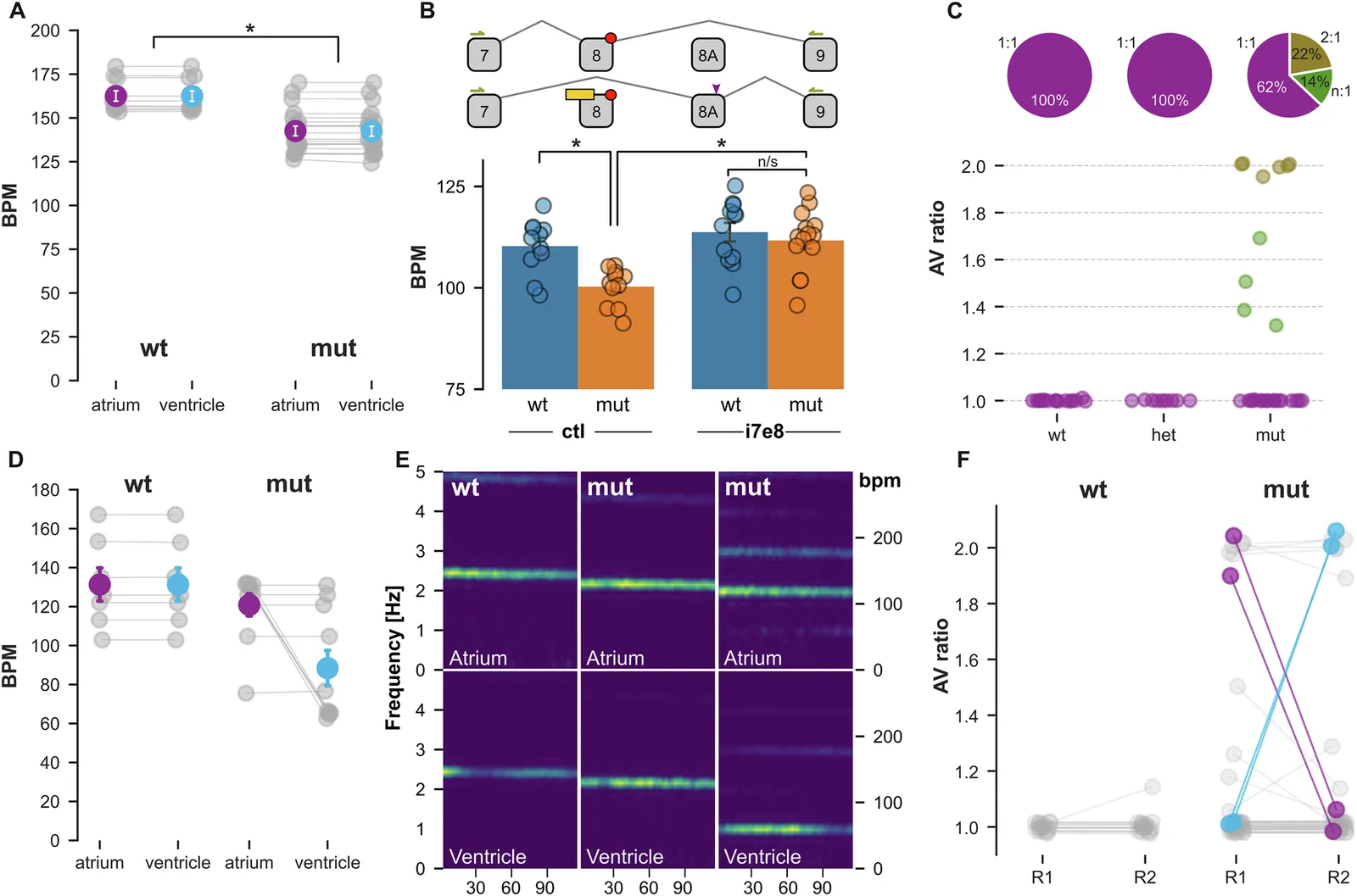
Embryonic bradycardia and intermittent arrhythmia phenotypes. **A** Contraction frequency for cardiac chambers in 2 dpf wildtype and mutant larvae (*N* = 10, 20). *t-test *p* < 0.001. **B** Antisense oligonucleotide (yellow) used to remove the exon 8 mutation (red) by shifting isoform expression from exon 8 to exon 8A. Arrows, RTPCR primers. Arrowhead, EcoRI site. Atrium contraction frequency at 2 dpf in larvae injected with standard control (ctl) or i7e8 splice-blocking morpholino (*N* = 7–10/group). Mutants showed bradycardia (ANOVA, main effect of genotype, F_1,30_ = 6.6, *p* = 0.015). n/s, not significant. **C** Atrium:Ventricle contraction frequency ratio (AV ratio) for wild-type, heterozygous and mutant larvae (*N* = 16, 9, 27). Piecharts: percent of larvae for each genotype with a 1:1, 2:1 or intermediate (n:1) ratio. **D** Contraction frequencies in 5 dpf larvae (wild-type, *N* = 7; mutant, *N* = 10). Mean, standard error indicated. **E** Spectrograms for 5 dpf larvae showing power at 0–5 Hz frequencies (right axis, 0–300 bpm). Top panels, atrium. Bottom panels, ventricle. **F** AV ratio for wild-type and mutant (*N* = 17, 41) larvae recorded twice with at least a 4 h interval between the first (R1) and second (R2) recording. Larvae that switched between AV ratios highlighted by colored lines.

Life-threatening episodes of arrhythmia (including long QT-interval, bradycardia and 2:1 atrioventricular block) are a characteristic feature of Timothy syndrome. The loss-of-function *cacna1c* mutation (*island beat*) had dissimilar effects on muscle cells from two cardiac chambers in zebrafish, with atrial cardiomyocytes showing uncoordinated contractions and ventricular cardiomyocytes failing to contract, even in response to direct electrical stimulation^12^. We therefore used high-speed video recordings to monitor each cardiac chamber separately. Contraction frequency was normal in 2 dpf heterozygous embryos (Supplementary Fig. S4A) but was significantly reduced in both chambers in homozygotes (Fig. 3A).

The expression of alternative splice isoforms of *cacna1c* has been leveraged to correct deficits in a human TS1 organoid model^36^. An antisense oligonucleotide that prevented splicing of exon 8A, which included the gain of function mutation, led to increased expression of the unaffected exon 8 and rescued deficits in neural development. We designed an antisense morpholino to block splicing from exon 7 to exon 8 at the intron 7–exon 8 (i7e8) junction, leading to increased inclusion of exon 8A (Supplementary Fig. S4B). Embryos injected with the i7e8 morpholino showed increased cardiac contraction frequency (ANOVA main effect of treatment, F_1,44_ = 16.7, *p* < 0.001 ; Fig. 3B, Supplementary Fig. S4C, D), such that mutants treated with the i7e8 morpholino showed a contraction frequency similar to wild-types and were therefore normalized.

By 3 dpf, we noted that mean atrial contraction rates in homozygotes were no longer significantly reduced, but that in some larvae, the ventricle contraction frequency was half that of the atrium, a phenomenon seen in 2:1 atrioventricular (AV) block (Fig. 3C–E, Supplementary Video 2, 3). Whereas all wild-type and heterozygous larvae showed matched (1:1) AV contraction frequency, 22% (6/27) of mutants had a 2:1 rhythm, and 14% an intermediate ratio (Fig. 3C). To assess whether arrhythmia was intermittent or a stable phenotype in individual mutant larvae, we recorded from the same larvae twice, at an interval separated by 4–6 h. Around 10% (4/41) of mutants switched between matched AV rhythms and a 2:1 beat frequency (Fig. 3F). We observed an abrupt switch from a matched AV rhythm to a 2:1 rhythm during one recording (Supplementary Fig. S4E). Thus, homozygous mutants have bradycardia at early stages, later followed by intermittent cardiac arrhythmia, consistent with findings in Timothy syndrome patients.

### TS2 larvae display elevated seizure susceptibility

**Fig. 4:**
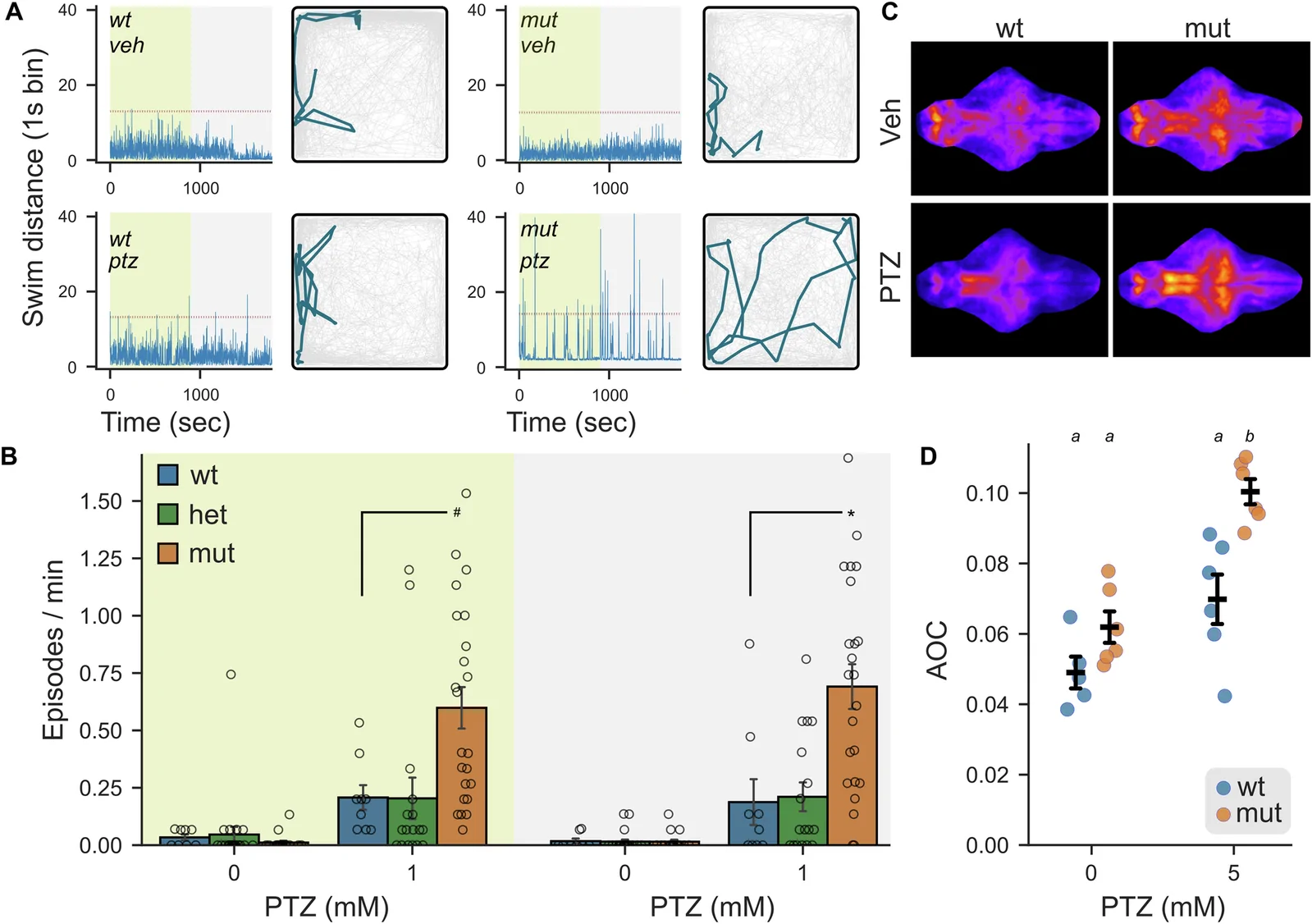
Increased seizure susceptibility in pentylenetetrazole sensitized mutants. **A** Locomotor activity in individual wild-type and mutant larvae, under illuminated (yellow background) or dark (gray background) conditions, after exposure to 1 mM PTZ (ptz) or vehicle (veh). Y-axis represents swim distance (total pixel movement in 1 s recording intervals). Red dotted line indicates distance threshold for designating seizure-like episodes. Traces to the right of each plot show the position of the larva over a 10 s interval during the light phase. **B** Seizure-like movement episodes per minute in wild-type, heterozygote and mutant TS2 larvae recorded for 15 min in illuminated (left) or dark (right) conditions with vehicle alone (*N* = 8 wild-type, 22 heterozygote, 18 homozygous mutants) or in 1 mM PTZ (*N* = 9 wild-type, 17 heterozygote, 23 homozygous mutants). #MWU test *p* = 0.01 and *p = 0.004, for mutants compared to wild-types. **C** Mean brain GCaMP epifluorescence intensity for larvae exposed to vehicle (wt, *N* = 5 ; mut, *N* = 6) or 5 mM PTZ (wt, *N* = 6 ; mut, *N* = 6) over a 20 min recording period. **D** Area under the curve (AOC) for GCaMP fluorescence intensity during recordings in (**C**). Mean and standard error indicated for each group. ANOVA between groups: F_3,19_ = 17.8, *p* < 0.001. Letters indicate groups that are significantly different in post-hoc Tukey tests.

As seizure disorders are seen in a majority of TS2 patients^5^, we assessed whether TS2 fish showed heightened seizure susceptibility. We exposed larvae to pentylenetetrazol (PTZ), a GABA receptor antagonist that, at low doses, only slightly elevates the risk of seizure-like activity in larval zebrafish^37^. We scored seizures by detecting large-amplitude swimming movements that were detected at a frequency of less than one event per 15 min recording period in untreated wildtype larvae (Supplementary Fig. S5A, B). There were no genotype-related differences in locomotor activity in larvae not exposed to PTZ (Fig. 4A, B, PTZ 0 mM). However, after PTZ treatment, homozygous mutants tended to show long periods of immobility interspersed with unusually vigorous swim bouts, similar to previous descriptions of seizure-like activity in zebrafish (Fig. 4B, PTZ 1 mM). We also imaged brain activity using pan-neuronal GCaMP expression in immobilized larvae during PTZ treatment (Fig. 4C). Brain activity showed a much stronger elevation in homozygous mutants than in wildtype siblings, consistent with greater susceptibility to seizures (Fig. 4D). “Frequent startle reflexes” and distress caused by loud noises were reported for TS2 patients^3,5^. While the exact cause of these auditory-associated symptoms in TS2 are unknown, we tested auditory sensory and sensorimotor gating but did not detect changes in escape reactivity or prepulse inhibition (Supplementary Fig. S5C–E). Thus, homozygous mutant zebrafish share seizure susceptibility with patients but not increased auditory reactivity.

### Altered brain morphology and neuronal distribution in TS2 larvae

To examine brain structure changes in TS2 mutants systematically, we measured brain structure and composition using voxel-based morphometry^14^. For morphometric analysis, we imaged wild-type and mutant sibling larvae, each carrying two transgenes: the *tuba:mCardinal* transgene that labels all post-mitotic neurons, and the *gad1b:RFP* transgene that labels GABAergic neurons. We then used elastic registration to align confocal scans to the Zebrafish Brain Browser (ZBB) reference brain^38,39^. Next, we computationally extracted clusters of significantly different voxels in the resulting transformation matrices to assess local changes in brain volume and clusters of voxels with altered fluorescence intensity to locate changes in *tuba:mCardinal* or *gad1b:RFP* signals^14^.

**Fig. 5:**
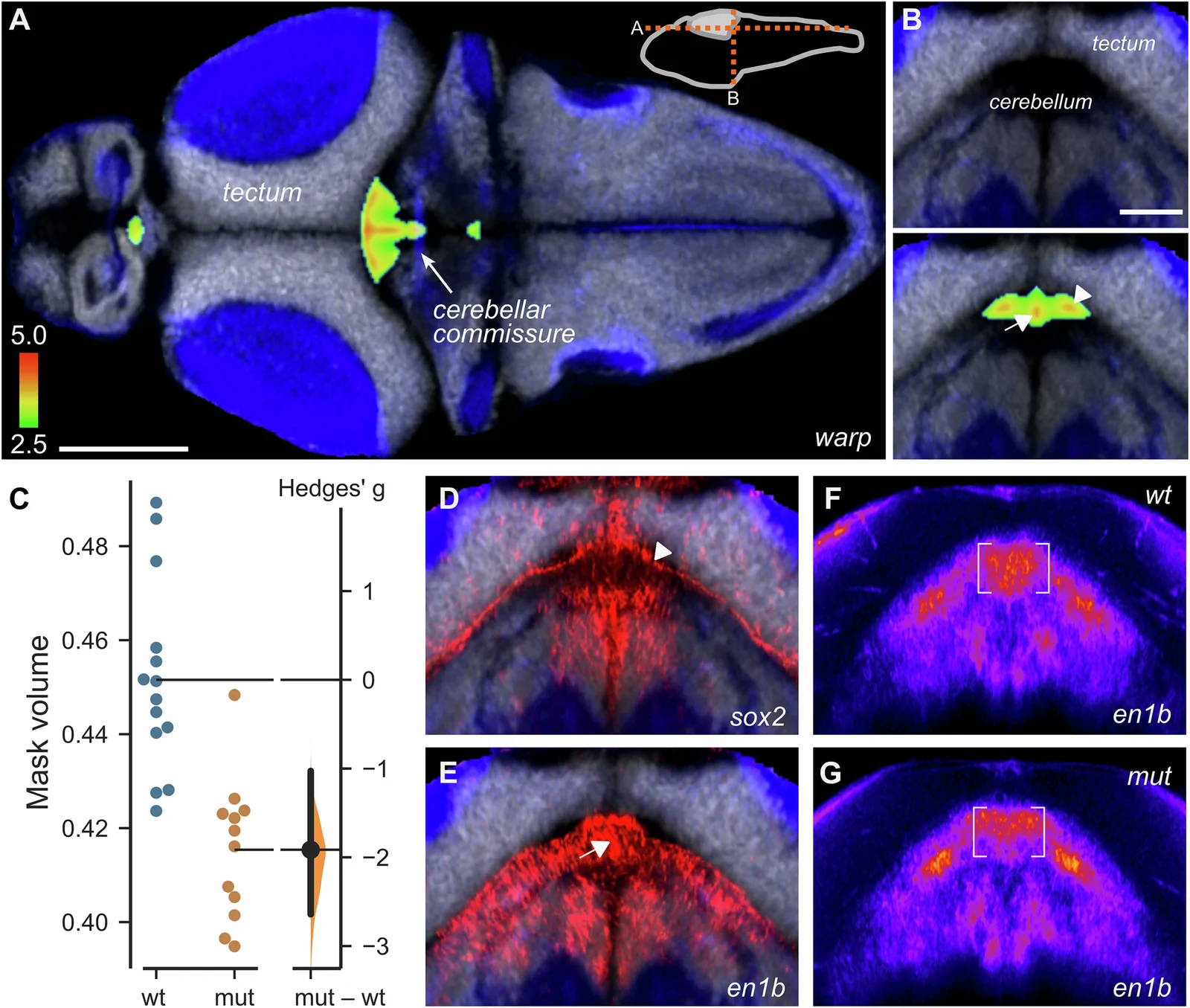
Decreased midbrain-hindbrain boundary. Voxel clusters disproportionately reduced in volume in homozygous mutants compared to wild-type siblings, shown in horizontal (**A**) and coronal (**B**) sections through the brain at the levels indicated in the inset diagram. Cellular (gray, *huc:h2b-GFP*) and neuropil rich (blue, *huc:lyn-RFP*) regions annotated using images in ZBB. Color scale shows the nominal p-value for voxels that were part of statistically significantly affected clusters. **C** Volume (expressed as percent of total brain volume) of three-dimensional mask comprising the region of compressed voxels in (**A**), in an independent cohort of wild-type and mutant larvae. Estimation graph includes Hedges’g as a measure of the effect size for the difference in means between the groups, showing that the decrease in brain volume in mutants is significant. HCR fluorescent in situ hybridization for *sox2* (**D**) and *en1b* (**E**), at the same coronal section as in (**B**). Images are the mean of co-registered confocal scans from multiple larvae. Arrows in (**D**) and (**E**) are aligned with B. In situ hybridization for *en1b* in wild-type (**F**) and mutant (**G**) larvae at the same coronal plane as in B. Each image is the average of 9 larvae after registration to ZBB using affine-only parameters (to prevent warping from obscuring differences). Fluorescence intensity pseudocolored to aid comparison.

Mean brain volume was similar in wild-type and heterozygous sibling 6 dpf larvae, but reduced by 6.1% in homozygous mutants (F_2,64_ = 4.3, *p* = 0.018; Supplementary Fig. S6A); the size reduction was greater for brain regions primarily occupied by neuronal somas than for primarily neuropil regions (F_2,65_ = 7.2, *p* = 0.001; Supplementary Fig. S6B). Analysis of cell nuclei density in several brain regions revealed a significant reduction in the stratum periventriculare of the optic tectum, a cell dense region in the midbrain (Supplementary Fig. S6C,D). Voxel-wise analysis revealed a salient cluster of voxels that were disproportionately compressed in a midline region in the anterior hindbrain, located between the cerebellar commissure and the caudal lobe of the optic tectum (Fig. 5A). This region is occupied neither by the somas of mature neurons nor by neuropil (Fig. 5B). To assess the reproducibility of this finding, we annotated the reference brain with a mask that covered the affected region, then imaged an independent cohort of larvae. The volume occupied by the mask was reduced by 8% in mutants compared to wild-types (even after normalizing for the smaller brain volume in mutants), confirming a change in brain architecture in this region (t-test, *p* < 0.001 ; Fig. 5C). Heterozygotes also showed a 6.7% reduction in the same region (t-test, *p* < 0.001; Supplementary Fig. S6E). The ZBB atlas revealed that the affected region is bounded by expression of *sox2*, a marker of neural stem cells, and overlaps with *en1b*, which labels the midbrain-hindbrain boundary (Fig. 5D, E). In situ hybridization showed that the extent of *en1b* expression was reduced in mutants, overlapping with the region of reduced volume (Fig. 5F, G), whereas *sox2* expression remained unchanged (Supplementary Fig. S6F). Thus, the midbrain-hindbrain boundary region is disproportionately reduced in size in homozygous mutants.

**Fig. 6:**
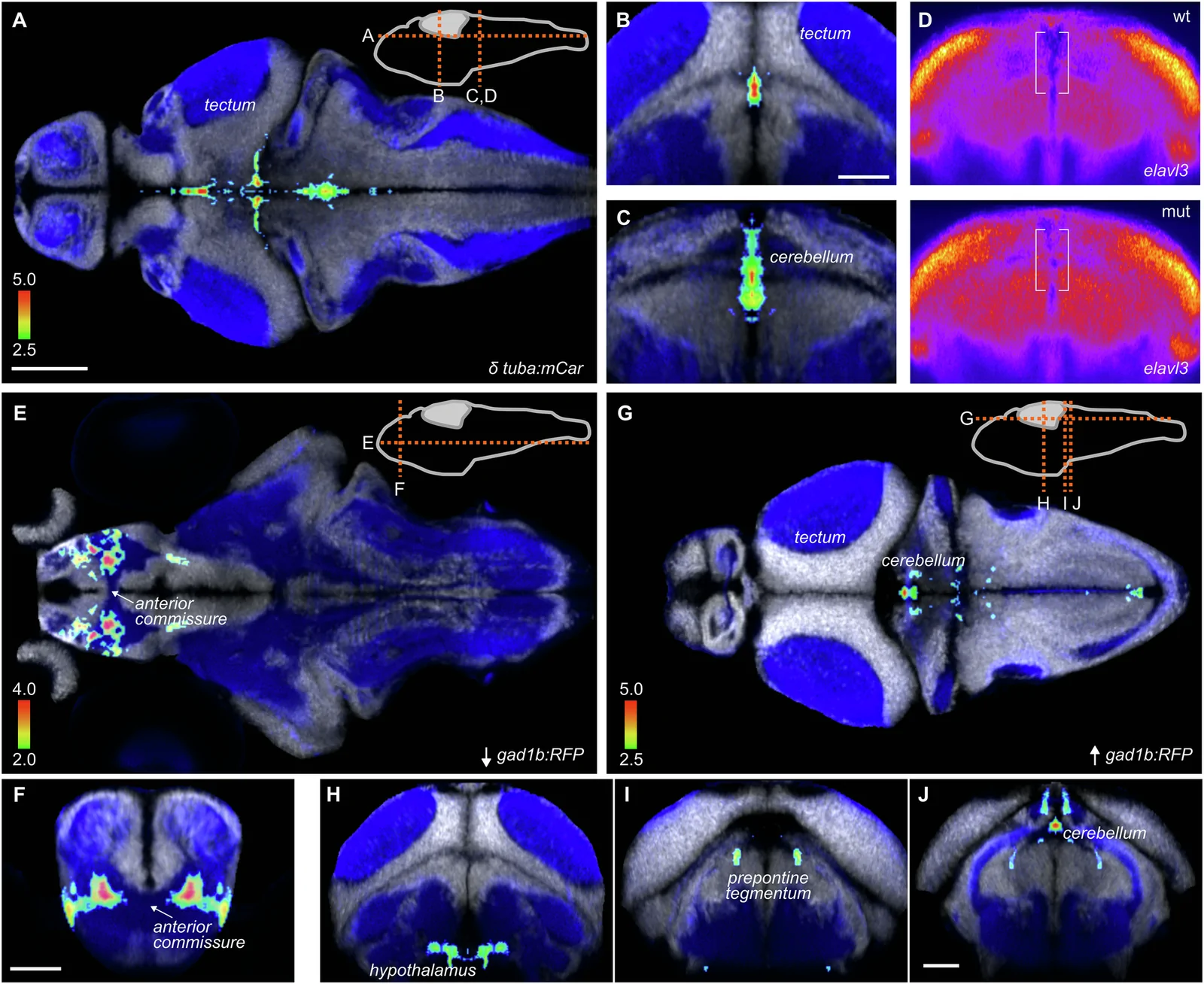
Changes in neuron distribution in TS2 homozygous larvae. Increased *tuba:mCardinal* expression in homozygous mutants, shown in horizontal (**A**) and coronal (**B**, **C**) slices, shown at the level indicated in the schematic in (**A**). Color scale shows nominal p-value for each voxel with increased expression in mutants. Background showing cell soma rich areas (*elavl3:h2b-GFP*, white) and neuropil (*elavl3:lyn-RFP*, blue) from co-registered brain scans in ZBB, taken from MapZeBrain^74^. **D** Fluorescent in situ hybridization for *elavl3* in wild-type and mutant TS larvae. Each image is the mean of 9 brain scans, co-registered using affine scaling only. Coronal re-slice of confocal images shown is a 10 µm projection corresponding to the level in B. Clusters of voxels with decreased (**E**, **F**) and increased (**G**–**J**) *gad1b:RFP* signal in mutants. Panels in (**F** and **H**–**J**) show coronal re-sections at levels indicated in schematics (**E**) and (**G**), respectively. Scale bars: 100 µm.

In these experiments, we unexpectedly noted an increase in the *tuba:mCardinal* signal in a midline region throughout the midbrain and anterior hindbrain that normally does not contain differentiated neurons (Fig. 6A–C). To substantiate this, we designed a mask encompassing the area of increased signal in the anterior hindbrain, and measured *tuba:mCardinal* signal intensity in independent cohorts of wild-type, heterozygous and mutant larvae. Increased midline fluorescence signal was present in both mutant and heterozygous TS2 animals compared to their wild-type siblings (Supplementary Fig. S7A, B). We also performed fluorescent in situ hybridization for *elavl3*, a second marker of post-mitotic neurons. Mutants showed excess *elavl3* signal in the same midline region (Fig. 6D). Tissue distortion during processing required for in situ hybridization impeded quantitative replication, however a meta-analysis of three experiments confirmed that there was a significant increase in *elavl3* signal (*p* = 0.002; Supplementary Fig. S7C, D). Thus, homozygous mutants inappropriately express markers associated with mature neurons in a brainstem midline region that is normally occupied by neural stem cells and neuroblasts.

Next, we analyzed the *gad1b:RFP* signal in mutants to examine the distribution of inhibitory neurons. Mutants showed a 5% reduction in mean fluorescence intensity in the subpallium, an effect that was also observed in a second cohort of larvae, but not in heterozygotes (Supplementary Fig. S7E–G). We then combined the two cohorts by performing an ANOVA at each voxel. This disclosed that voxels with the greatest decrease in RFP signal were located in a subpallial neuropil area adjacent to the anterior commissure (Fig. 6E, F), and revealed small clusters of increased *gad1b:RFP* fluorescence intensity in the cerebellum, prepontine tegmentum and hypothalamus (Fig. 6G–J).

To help interpret these findings, we analyzed the expression of *cacna1c* during neural development. Single-cell sequencing data from Daniocell, a publicly available dataset generated from staged zebrafish embryos, showed that *cacna1c* expression increases in developing brain starting at around 36 hpf, closely paralleling the expression of *synaptophysin a* (*sypa*), a marker of mature neurons (Supplementary Fig. S8)^19^. We used fluorescent in situ hybridization to examine *cacna1c* mRNA distribution in developing brain. Expression of *cacna1c* was detected in the telencephalon, optic tectum, thalamus and rhombencephalon, although not adjacent to midline proliferative zones, at 2 dpf (Supplementary Fig. S9A) and persisted in these regions at 3 dpf (Supplementary Fig. S9B). Bands of *cacna1c*-expressing cells in the hindbrain ran parallel to the brain axis and were especially distinct in coronal views. Cell groups with especially high expression of *cacna1c* were found in the cerebellum, where an emerging cluster was visible at 3 dpf. At 6 dpf, *cacna1c* continued to show widespread expression in the pallium, subpallium, diencephalon and optic tectum, with a stronger signal in pallium, subpallium and cerebellum (Supplementary Fig. S9C–E). There were two strong distinct bilateral patches of expression within the cerebellum: a rostromedial cluster located in the teleost-specific valvula cerebelli and a caudolateral cluster that overlapped with co-registered scans for *gad1b*, a marker for inhibitory neurons, and *pvalb6*, a gene highly expressed in mature Purkinje neurons^40^ but not with *neurod1*, a marker for granule cells (Supplementary Fig. S9F). Expression of *cacna1c* in 3 dpf and 6 dpf brains did not overlap with areas of highest *elavl3* expression, but rather with the adjacent areas where *elavl3* was less robustly expressed (Supplementary Fig. S9G, H). Neurons strongly express *elavl3* immediately after becoming post-mitotic, and then expression decreases to intermediate levels in mature neurons. Thus, in the developing zebrafish brain, *cacna1c* expression level in mature neurons is higher than in proliferating cells or newly generated neurons.

### Elevated temperature exacerbates phenotypes in TS2 mutants

**Fig. 7:**
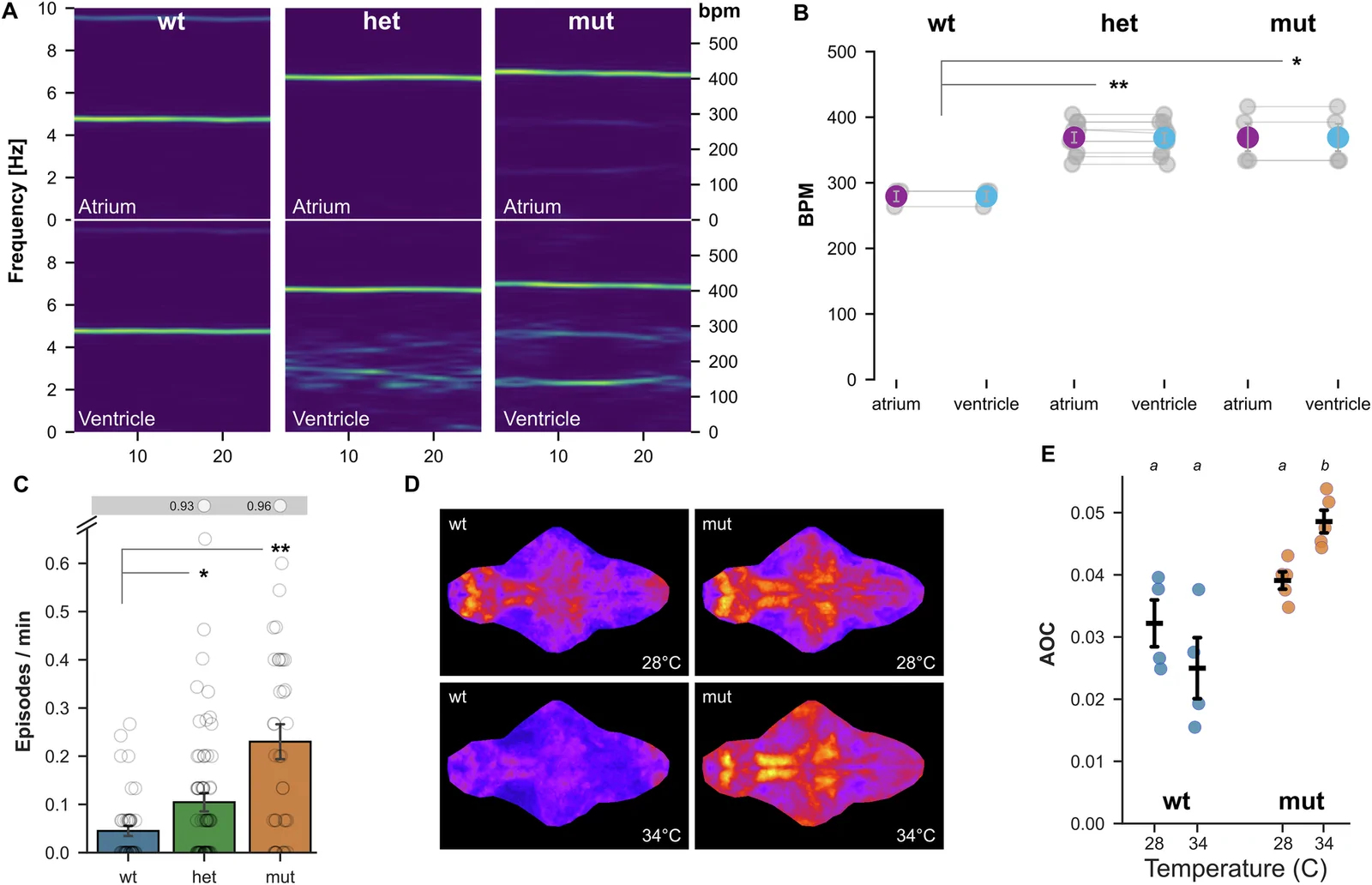
Elevated temperature triggers arrhythmia and seizure-like behavior in TS2 mutants. **A** Spectrograms of cardiac contraction frequencies in wild-type, heterozygous and mutant atria (top panels) and ventricles (bottom panels), when tested in the center of an arena warmed to 37 °C. **B** Atrium and ventricle contraction frequency for wild-type, heterozygous and mutant larvae (*N* = 3, 10, 4, respectively). F_2,14_ = 12.3, *p* < 0.001. post-hoc Tukey test, **p < 0.001 and *p = 0.003. **C** Episode frequency of seizure-like movement for wild-type, heterozygous and mutant larvae (*N* = 44, 73, 37, respectively) at 37 °C. Results are for three experiments combined: see Supplementary Fig. 10E, F for experiments individually. Kruskal–Wallis H_2_ = 21.9, *p* < 0.001. Dunn’s post-hoc comparison, **p < 0.01 and *p < 0.05. **D** Mean GCaMP fluorescence for wildtype and mutant larvae at 28 °C and 34 °C. **E** Area under the curve (AOC) for GCaMP fluorescence intensity in (**D**). Mean and standard error indicated (*N* = 4,5 larvae for wild-type and homozygous mutants). Repeated measures ANOVA interaction between temperature and genotype: F_1,7_ = 18.2, *p* = 0.004. Letters indicate groups that are significantly different in post-hoc comparisons.

Case reports suggest that individuals with long QT syndrome (including in one TS2 case) may be especially at risk for severe arrhythmias during febrile illness^8,11^. Opening of Ca_V_1.2 calcium channels at hyperpolarized membrane potentials when the temperature is above 37 °C may contribute to this pathology^10^. We reasoned that individuals with even one TS2 gain of function allele might be highly sensitive to increased temperatures, potentially explaining why heterozygous mutations have such severe consequences in mammals, whereas heterozygous zebrafish (which are maintained at 28 °C) showed relatively subtle changes. We speculated that we might reveal phenotypes in heterozygous larvae by shifting them to 37 °C, a temperature that is normally well tolerated by zebrafish. Consistent with a previous report, cardiac contraction frequency in 5 dpf wild-type larvae increased to around 290 beats per minute (bpm) at 37 °C (Fig. 7A^41^. Atrioventricular contraction rates remained coupled and spectrograms revealed no additional frequencies. However, heterozygous and mutant larvae showed significantly elevated contraction frequencies compared to wild-types, and ventricle spectrograms revealed a range of additional frequencies suggesting arrhythmia (Fig. 7A, B; Supplementary Fig. S10A,B). Heart rate in larvae of all genotypes exposed to 37 °C for 30 s decreased after 90 min of recovery at 28 °C, but remained slightly elevated (Supplementary Fig. S10C), Thus, as hypothesized, at a temperature expected to activate L-type calcium channels, heterozygous larvae exhibited arrhythmia, whereas at lower temperatures, they resembled wild-type larvae.

We asked whether heat sensitivity extended to the manifestation of seizure-like activity. At 28 °C, larvae that were not treated with pentylenetetrazol showed very few seizure-like locomotor episodes, irrespective of genotype (Fig. 4B). In contrast, at 37 °C, both heterozygous and homozygous mutants showed a significantly greater frequency of seizure-like episodes than wild-types, even in the absence of sensitization with pentylenetetrazol (ANOVA main effect of genotype F_2,145_ = 17.2, *p* < 0.001; Fig. 7C). The increase in seizure-like episodes was not part of a generalized increase in movement: the swimming speed during movement bouts was not significantly different between mutation carriers and wild-type embryos (F_2,145_ = 0.88, *p* = 0.42; Supplementary Fig. S10D). Elevated seizure-like behavior was greater and more robust across experiments in homozygous than heterozygous larvae (Supplementary Fig. S10E, F). We next examined how elevated temperature effected neural activity in mutants. As previously reported, GCaMP fluorescence was reduced at increased temperature (ANOVA, main effect of temperature, F_1,7_ = 16.5, *p* = 0.005; Supplementary Fig. S11)^42^. However, at elevated temperature, activity transients were significantly increased in homozygotes compared to 28 °C, corroborating the idea that the mutant channel causes increased heat sensitivity (Fig. 7D, E). Together with the appearance of cardiac and behavioral phenotypes in heterozygous larvae when tested at an elevated temperature, these results suggest that normal rearing temperatures protect larvae against adverse effects of the *cacna1c* gain of function mutation and support the idea that fever may be a preventable risk in TS.

## Discussion

Acute stressors, particularly hypoglycemia and respiratory distress, have been linked to serious adverse outcomes in TS^5^. However, risks associated with fever, which causes lethal arrhythmias in other long QT syndromes, are unknown. We generated a new zebrafish model with the homologous gain-of-function mutation to assess TS pathophysiology. Cacna1c is highly conserved; in particular, the 30 amino acid residues that flank the mutation site are identical in human and zebrafish. Cacna1c^G419R^ zebrafish recapitulate key features of Timothy syndrome: bradycardia, episodic arrhythmia and seizure susceptibility. Homozygous mutant TS2 zebrafish showed a reduction in the midbrain-hindbrain boundary and, correspondingly, a reduction in the size of adjacent midbrain and hindbrain areas. Several neuropeptide genes showed elevated expression in mutants, including *bdnf* and *vgf*, genes implicated in the control of neurogenesis. Although TS is a dominant disorder, heterozygous zebrafish showed only subtle abnormalities in brain morphology and transcription but normal survival and fertility. Under standard conditions, heterozygotes did not show arrhythmia or seizure susceptibility, but produced these phenotypes when body temperature was raised to 37 °C, a condition where Ca_V_1.2 channels open spontaneously^10^.

Signature features of TS include a prolonged QT interval, syndactyly and neurodevelopmental abnormalities including autism and seizures^3^. TS2, which arises from a G406R mutation in exon 8 rather than exon 8A, is clinically similar but without syndactyly. To assess the extent to which deficits in Cacna1c^G419R^ zebrafish resemble clinical manifestations in TS2, we focused on cardiac and seizure phenotypes, which have been extensively characterized in other zebrafish models. TS2 mutant fish exhibited bradycardia at 2 dpf and intermittent arrhythmia similar to 2:1 atrioventricular block (AVB) as older larvae. Bradycardia and AVB are present in a majority of TS2 patients perinatally^5^. Homozygous (but not heterozygous) TS2 larvae exposed to a low dose of the GABA receptor antagonist pentylenetetrazol showed an increase in the number of seizure-like locomotor events compared to siblings. Together, the presence of specific cardiac arrhythmias – bradycardia and 2:1 AVB – and seizure susceptibility indicate that TS2 larvae recapitulate key TS clinical manifestations and may therefore provide a useful tool for investigating how gain-of-function mutations in this disorder disrupt normal development.

During neural development, there is a switch from the inclusion of exon 8A in neural progenitors to the inclusion of exon 8 in mature neurons^4,6^. It was recently shown that the use of an antisense inhibitory oligonucleotide to prevent the inclusion of exon 8A increased exon 8 inclusion and rescued neurodevelopmental phenotypes in a TS1 brain organoid model^36^. Injection of morpholino antisense oligonucleotides into zebrafish embryos is routinely used to block pre-mRNA splicing^43^, but it was not clear whether a similar strategy would be effective in zebrafish TS2 mutants where the mutation affects exon 8, which shows more than 10-fold greater utilization than exon 8A throughout development. However, a morpholino against the intron 7 - exon 8 boundary was highly effective in switching isoform expression from exon 8 to exon 8A. Cardiac contraction frequency in exon 8A-containing larvae was increased irrespective of genotype, such that mutants injected with the splice-blocking morpholino showed similar contraction frequency to wild-types injected with a control morpholino. This illustrates the utility of the zebrafish model for the development of antisense oligonucleotide-based therapeutics, where the external development and optical transparency of early larvae can enable studies of oligonucleotide effects on various cell types and help in delineating the optimal developmental window for treatment.

Consistent with a key role for intracellular calcium signaling in regulating gene expression in neurons^44,45^, gene expression in neurons derived from Timothy syndrome patient stem cells and stem cells from healthy controls differed considerably^46^. Accordingly, RNA-seq data from homozygous mutants showed widespread changes, prominently in the expression of genes that encode neuropeptides. A known downstream target of calcium influx through *cacna1c* containing L-type calcium channels is *bdnf* ^47^. RNA-seq revealed elevated expression of several neuropeptides in mutants, including *bdnf*, its downstream factor *vgf* and *si:dkey-175g6.2*, a second homolog of *vgf* present in zebrafish, which was also elevated in TS2 heterozygous larvae. Increased expression of these neuropeptides may contribute to changes in neurogenesis: overexpression of *Bdnf* in mouse visual cortex led to accelerated maturation of GABAergic innervation; a similar process could account for the elevated GABAergic signal observed in some brain regions of homozygous mutants^48^. Mice that overexpressed *Vgf* showed microcephaly and impaired cerebellum development^49^. Associations with differences in the size of brain or its divisions were reported for several other genes differentially regulated in homozygous mutants. Elevated expression of *tent5d* (also known as *fam46d*) and reduced expression of *mcm7* and *bub1* orthologs have been reported in mice and human patients with microcephaly^50–52^. We also observed decreased expression of *gcgb* (*glucagon b*) in mutants, consistent with reports of lower glucagon immunoreactivity and impaired glucagon secretion during hypoglycemia in a TS mouse model and possibly contributing to life-threatening hypoglycemia episodes in patients^7^. Thus, *cacna1c* may have a conserved role in regulating energy metabolism in vertebrates.

We found that *cacna1c* is broadly expressed throughout stages of neural development in zebrafish, with conspicuous expression in the cerebellum. The relative expression levels of *cacna1c* over time paralleled that of the mature neuron marker, *sypa*; however, other evidence suggests that *cacna1c* may also regulate early developmental processes. Brain size was diminished in mutants, and in particular the total volume of brain regions that are predominantly cellular was reduced compared to the total volume of neuropil-rich regions. There was a disproportionately large reduction at the midbrain-hindbrain boundary (MHB) and a matching loss of *en1b* expression, a MHB marker. The MHB controls the development of the adjacent cerebellum and optic tectum, and consistent with this, we saw a significant reduction in cell density in the major neuron-containing area of the optic tectum, the stratum periventriculare. In parallel, we observed an increase in the expression of two mature neuron markers – the transgenic line *tuba:mCardinal*, and in situ hybridization for *elavl3* – adjacent to the brain midline in mutants, a region that normally includes stem cells and proliferating neuroblasts. L-type calcium channels have been implicated in regulating neuronal proliferation^53^. In humans, *CACNA1C* is strongly expressed in the outer subventricular zone, where it may contribute to the depolarizing effects of GABA on neural progenitor cells and regulate cell fate decisions^4^. Premature terminal differentiation of proliferating neuroblasts might explain the smaller overall brain volume in mutants, with increased neuron signal at the midline. Indeed, *bdnf* and *vgf*, genes that were upregulated in homozygous mutants, are known to regulate neurogenesis^34,35^. However, LTCCs are also known to regulate neuronal migration^54^. TS2 mice showed an increase in tangential migration of inhibitory neurons from the median ganglionic eminence (MGE)^55^. Conversely, electroporation of *Cacna1c* mRNA with the TS mutation suppressed the radial migration of excitatory neurons into the upper cortical layers in mice^56^, and in an organoid model, neurons from TS1 patient-derived stem cells showed delayed migration of MGE interneurons^36,57,58^. On the other hand, neither inhibition nor activation of L-type calcium channels affected the migration of newborn neurons in the olfactory bulb of postnatal mice^59^. The effects of LTCC activity on neuronal migration appear to vary with stage and cell type in the developing brain, and thus, the expression of mature neuron markers at the midline region in TS2 mutants might reflect premature terminal differentiation of neuroblasts into neurons, suppressed neuronal migration, or both.

Multiple zebrafish models exhibiting seizure-like behavior have been previously characterized (for example^60–62^). However, only a minority of zebrafish genetic models of epilepsy disorders show seizure-like swimming behavior; rather neural hyperexcitability may be a more reproducible proxy for human seizure disorders^63–65^. Accordingly, TS2 larvae exhibited seizure-like swimming when sensitized with PTZ and elevated baseline neural activity. Zebrafish have also been widely used to study cardiac arrhythmia, including analysis of mutants that like the homozygous mutant, manifest atrioventricular block^66,67^.

Significant cardiac and behavioral changes were detected only in homozygous mutant fish, which could be raised with only minor loss of viability to fertile adults. In contrast, patient mutations are dominant and lethal. It is not uncommon for mutations that cause haploinsufficient psychiatric disorders to be homozygous viable in zebrafish^68^, however, given the severe effects in TS patients we considered whether phenotypic effects of the mutation were compensated or masked in zebrafish. It is unlikely that a redundant gene mitigates *cacna1c* mutations because inactivating mutations in *cacna1c* cause a lethal impairment of heart ventricle development in zebrafish^12^. Patient mutations affect the second to last nucleotide of exon 8 or 8A, and in an induced pluripotent stem cell model, this disrupts splicing leading to a shift in isoform use^4^. However, we did not detect differences in isoform expression or splice site selection in TS2 mutants. Of the 8 other L-type voltage-gated calcium channel genes in zebrafish, the only significant change at either 2 or 6 dpf was a 10% increase in expression of *cacna1sb* at 2 dpf, making it unlikely that genetic compensation explains the survival of homozygous mutants. We therefore think that cellular adaptation to the mutation is unlikely to explain why heterozygous zebrafish show only mild phenotypes. Rather, we speculate that biological and/or environmental factors may account for heterozygous viability. First, in the human heart, L-type calcium channels constitute more than 90% of all calcium channel transcripts, compared to only 35% in zebrafish, where the majority are T-type calcium channels^69^. Second, zebrafish ventricular myocytes primarily rely on extracellular calcium influx for contraction, whereas calcium-induced calcium release from the sarcoplasmic reticulum in human cardiomyocytes can amplify the effects of changes in extracellular calcium influx^70^. Third, it has been argued that due to intrinsic differences in the cardiac conduction system and repolarization properties in fish, delayed repolarization of cardiomyocytes is less likely to cause arrhythmia than in mammals^71^. These factors may reduce the sensitivity of zebrafish to activating mutations in *cacna1c*.

Notably, the mild phenotype of heterozygous larvae enabled us to directly test our central idea: whether elevated body temperature is a risk factor in TS. This hypothesis is based on the previously described temperature sensitivity of L-type calcium channels^10^. Electrophysiological experiments on ventricular cardiomyocytes and Ca channels expressed in Xenopus oocytes pointed at accelerated transitions between inactivated states, rather than channel activation or Ca-dependent inactivation, as the mechanism underlying the increased probability of open state for L-type calcium channels at elevated temperature^72,73^. Elevated temperature unmasked seizure-like behavior in heterozygous larvae, which were normal under standard rearing conditions. Moreover, in contrast to measurements at 28 °C where a phenotype occurred only in homozygous mutants, at 37 °C both mutant and heterozygous larvae showed atrial and ventricular tachycardia. As TS patients are prone to infections, elevated L-type calcium channel activity during fever may lead to tachyarrhythmia, which is a major cause of mortality, and in fact infection has been reported as an associated factor for sudden death in TS^5^. These findings emphasize the importance of studying whether patient outcomes may be improved by close monitoring and management of body temperature and provide a new model for studying the pathophysiology of TS and testing strategies that may mitigate risk associated with fever.

### Ethics approval

Animal experimental procedures were approved by the NICHD Animal Care and Use Committee and conducted in accordance with institutional guidelines (24.007).

## Supplementary information

Supplementary Information

Description of Additional Supplementary files

Supplementary Data 1

Supplementary Data 2

Supplementary Data 3

Supplementary Data 4

Supplementary Video 1

Supplementary Video 2

Supplementary Video 3

Reporting summary

## Data Availability

RNA sequencing data is available at the NCBI Sequence Read Archive repository (http://www.ncbi.nlm.nih.gov/sra/PRJNA1257950). Brain imaging data is available at Zenodo (http://zenodo.org/records/15325943). Other data needed to evaluate the conclusions in the paper are present in the paper or in Supplementary Data 4.
