# Supplementary material for: Elevated body temperature exacerbates arrhythmia and seizure-like activity in a zebrafish model of Timothy syndrome
Source: Commun Biol. 2026 Jul 9;9:1442. doi: 10.1038/s42003-026-10657-3 (PMC13724642; doi:10.1038/s42003-026-10657-3)
Supplement: Supplementary file 1 [file 42003_2026_10657_MOESM10_ESM.pdf]

nature portfolio

Corresponding author(s): Harold Burgess

Last updated by author(s): Jun 16, 2026

Reporting Summary

Nature Portfolio wishes to improve the reproducibility of the work that we publish. This form provides structure for consistency and transparency in reporting. For further information on Nature Portfolio policies, see our Editorial Policies and the Editorial Policy Checklist.

Statistics

For all statistical analyses, confirm that the following items are present in the figure legend, table legend, main text, or Methods section.

n/a	Confirmed	
☐	☒	The exact sample size (*n*) for each experimental group/condition, given as a discrete number and unit of measurement
☐	☒	A statement on whether measurements were taken from distinct samples or whether the same sample was measured repeatedly
☐	☒	The statistical test(s) used AND whether they are one- or two-sided<br>*Only common tests should be described solely by name; describe more complex techniques in the Methods section.*
☒	☐	A description of all covariates tested
☐	☒	A description of any assumptions or corrections, such as tests of normality and adjustment for multiple comparisons
☐	☒	A full description of the statistical parameters including central tendency (e.g. means) or other basic estimates (e.g. regression coefficient) AND variation (e.g. standard deviation) or associated estimates of uncertainty (e.g. confidence intervals)
☐	☒	For null hypothesis testing, the test statistic (e.g. *F*, *t*, *r*) with confidence intervals, effect sizes, degrees of freedom and *P* value noted<br>*Give P values as exact values whenever suitable.*
☒	☐	For Bayesian analysis, information on the choice of priors and Markov chain Monte Carlo settings
☒	☐	For hierarchical and complex designs, identification of the appropriate level for tests and full reporting of outcomes
☐	☒	Estimates of effect sizes (e.g. Cohen's *d*, Pearson's *r*), indicating how they were calculated

Our web collection on statistics for biologists contains articles on many of the points above.

Software and code

Policy information about availability of computer code

Data collection	Brain imaging: Nikon NIS elements software and Leica LAS X software for calcium imaging. Heartbeat video recordings: built-in software of Chronos camera. Behavior recordings: DAQ timer (National Instruments), built-in software of high-speed camera.
Data analysis	Statistical analysis of fish body size: JASP version 0.95.1. Brain image preprocessing: Image J (NIH; Schneider et al., 2012). Brain image registration: ANTS (Avants et al., 2008). Visualization of structural changes in the brain: Zebrafish Brain Browser (Marquart et al., 2016). Fish behavior analysis: Flote (Burgess and Granato 2007). Heart rate and rhythm analysis: sciPy package (Virtanen et al., 2020). Analysis of differential gene expression: FastQC v0.12.1, Cutadapt v 4.4, STAR v 2.7, Subread v 2.0.3, DESeq2 v1.33.0. Gene ontology analysis: ShinyGO (Get et al., 2020)

For manuscripts utilizing custom algorithms or software that are central to the research but not yet described in published literature, software must be made available to editors and reviewers. We strongly encourage code deposition in a community repository (e.g. GitHub). See the Nature Portfolio guidelines for submitting code & software for further information.

Data

Policy information about availability of data

All manuscripts must include a data availability statement. This statement should provide the following information, where applicable:

- Accession codes, unique identifiers, or web links for publicly available datasets
- A description of any restrictions on data availability
- For clinical datasets or third party data, please ensure that the statement adheres to our policy

RNA sequencing data is available at the NCBI Sequence Read Archive repository (https://www.ncbi.nlm.nih.gov/sra/PRJNA1257950). Brain imaging data is available at Zenodo (https://zenodo.org/records/15325943).

Research involving human participants, their data, or biological material

Policy information about studies with human participants or human data. See also policy information about sex, gender (identity/presentation), and sexual orientation and race, ethnicity and racism.

Reporting on sex and gender	Human subjects were not used in the study
Reporting on race, ethnicity, or other socially relevant groupings	Human subjects were not used in the study
Population characteristics	Human subjects were not used in the study
Recruitment	Human subjects were not used in the study
Ethics oversight	Human subjects were not used in the study

Note that full information on the approval of the study protocol must also be provided in the manuscript.

Field-specific reporting

Please select the one below that is the best fit for your research. If you are not sure, read the appropriate sections before making your selection.

☒ Life sciences ☐ Behavioural & social sciences ☐ Ecological, evolutionary & environmental sciences

For a reference copy of the document with all sections, see nature.com/documents/nr-reporting-summary-flat.pdf

Life sciences study design

All studies must disclose on these points even when the disclosure is negative.

Sample size	Selection of sample sizes was based on previous research done in the lab (Gupta et al., 2018, Burgess and Granato, 2007)
Data exclusions	No data were excluded from the analysis
Replication	Experiments were replicated in at least two independent cohorts of zebrafish larvae
Randomization	The study addressed the effects of the G419R mutation in the zebrafish cacna1c gene, and therefore allocation to groups was based on genotype, which was determined after the experiment in most cases. For morpholino oligonucleotide injections, fertilized eggs from the same clutch were collected and randomly assigned to control of splice-blocking oligonucleotide injection groups. For PTZ treatment, larvae were randomly assigned to PTZ or vehicle groups
Blinding	Investigators were blind to fish genotype in all experiments, except for imaging used for brain morphometry and heart rate recordings in morpholino oligonucleotide-injected larvae. Technical constraints precluded blinding in these experiments, but fish for recordings were drawn alternately from groups of different genotypes to minimize bias, and the analysis was performed automatically with the same software settings for all genotypes

Reporting for specific materials, systems and methods

We require information from authors about some types of materials, experimental systems and methods used in many studies. Here, indicate whether each material, system or method listed is relevant to your study. If you are not sure if a list item applies to your research, read the appropriate section before selecting a response.

Materials & experimental systems

n/a	Involved in the study
☒	☐ Antibodies
☒	☐ Eukaryotic cell lines
☒	☐ Palaeontology and archaeology
☐	☒ Animals and other organisms
☒	☐ Clinical data
☒	☐ Dual use research of concern
☒	☐ Plants

Methods

n/a	Involved in the study
☒	☐ ChIP-seq
☒	☐ Flow cytometry
☒	☐ MRI-based neuroimaging

Animals and other research organisms

Policy information about studies involving animals; ARRIVE guidelines recommended for reporting animal research, and Sex and Gender in Research

Laboratory animals	Larval zebrafish (aged 6 dpf) or younger of mixed TL/AB genetic background.
Wild animals	No wild animals were used in the study
Reporting on sex	Larval zebrafish were studied at the age of 6 dpf or younger, their sex is not determined at this age
Field-collected samples	No field-collected samples were used in the study
Ethics oversight	Animal care and experimentation protocols were approved by the Animal Care and Use Committee of Eunice Kennedy Shriver institute of Child Health and Human Development

Note that full information on the approval of the study protocol must also be provided in the manuscript.

Plants

Seed stocks	plants were not used in the study
Novel plant genotypes	plants were not used in the study
Authentication	plants were not used in the study
