# Supplementary material for: Elevated body temperature exacerbates arrhythmia and seizure-like activity in a zebrafish model of Timothy syndrome
Source: Commun Biol. 2026 Jul 9;9:1442. doi: 10.1038/s42003-026-10657-3 (PMC13724642; doi:10.1038/s42003-026-10657-3)
Supplement: Supplementary file 2 [file 42003_2026_10657_MOESM1_ESM.pdf]

Elevated body temperature exacerbates arrhythmia and seizure-like activity in a zebrafish model of Timothy syndrome

Svetlana A. Semenova, Amir M. Sams, Deepthi Nammi, Matthew Menold, Gennady Margolin, Jennifer L. Sinclair, Katelyn A. Robertson, Kerry Larkin, Sydney Kelly, Andy Golden, Benjamin Feldman, and Harold A. Burgess*

* burgessha@mail.nih.gov

Supplementary Information

Supplementary Figures 1 – 11

Supplementary Table 1

Supplementary Data 1. Differential gene expression analysis at 2 dpf comparing wild-type and homozygous mutant larvae. Comparison of all genes except for those on chromosome 4.

Supplementary Data 2. Differential gene expression analysis at 6 dpf. First sheet shows comparison for wild-type and homozygous mutant larvae. Second sheet shows comparison for wild-type and heterozygous larvae. Comparison of all genes except for those on chromosome 4.

Supplementary Data 3. Oligonucleotide sequences for HCR probes. Oligonucleotide sequences for probes used to perform HCR fluorescent in situ hybridization.

Supplementary Data 4. Numerical data underlying graphs and charts.

Supplementary Video 1. Elevated brain activity in mutants. Fluctuating GCaMP fluorescence signal in example wild-type (top) and homozygous mutant (bottom) larvae recorded over 5 min.

Supplementary Videos 2-3. Atrium and ventricular contractions. Videos showing atrial (blue outline) and ventricular (orange outline) contractions in wild-type (S2) and homozygous mutant (S3) larvae, slowed to half speed.

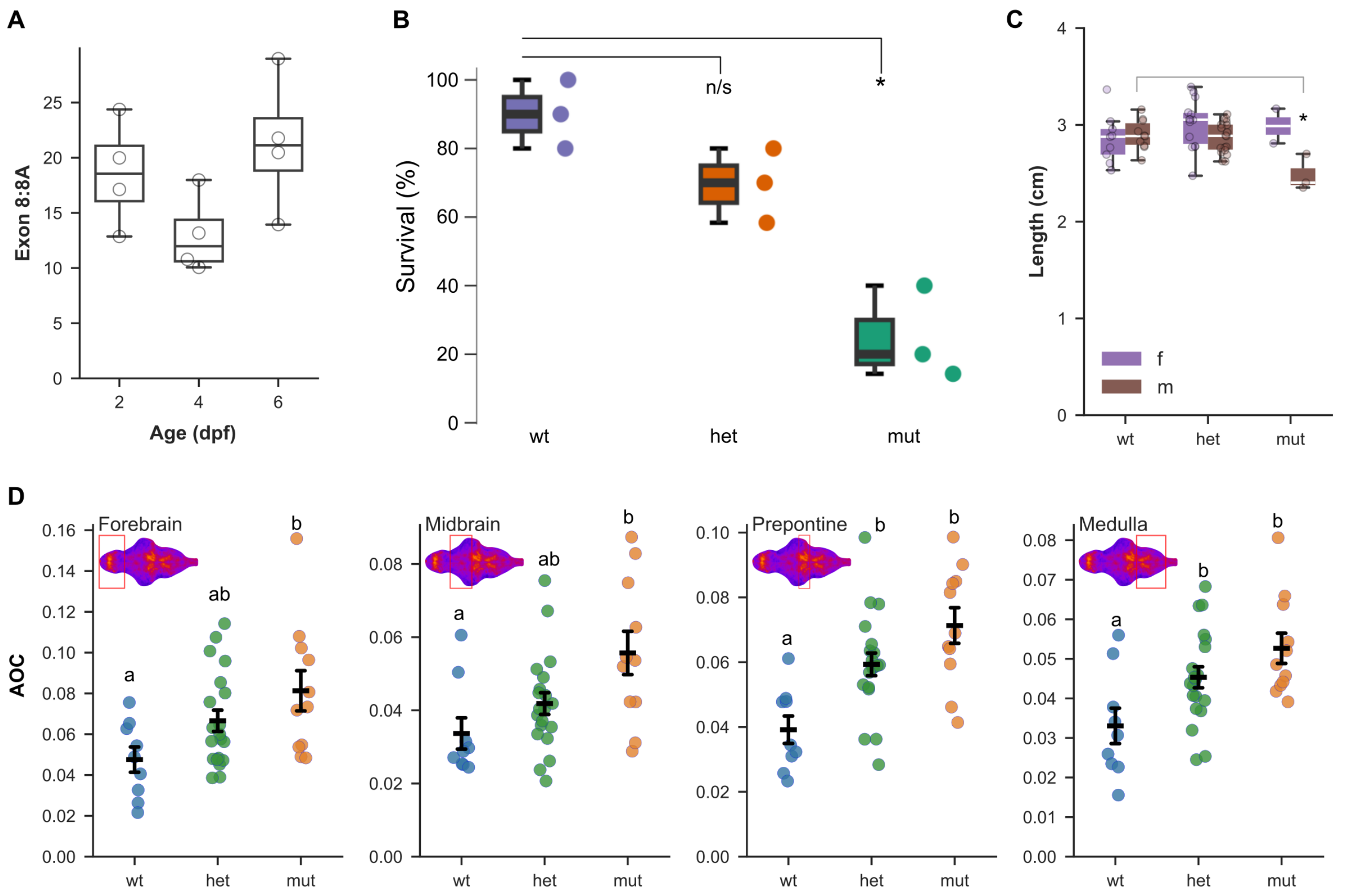

Supplementary Figure 1. Characterization of TS2 zebrafish. (**A**) Ratio of exon 8 to 8A expression in 2, 4 and 6 dpf larvae (qPCR). (**B**). Survival by genotype for sibling larvae raised in mixed groups of 20–30 individuals. N = 3 groups per genotype. ANOVA $F_{2,6} = 25.04$, $p = 0.001$. * post-hoc Tukey's test, $p = 0.001$. (**C**). Length of adult fish by genotype and sex (m, male ; f, female). ANOVA (genotype/sex interaction) $F_{2,50}=3.5$, $p = 0.037$. * post-hoc Tukey's test, $p < 0.05$. For box and whisker plots, box shows the 25-75 percentile range, and whiskers show 1.5x the interquartile range. (D) Area under the curve (AOC) for GCaMP signal in major brain divisions (indicated in schematic). ANOVAs: $F_{2,37} = 4.4$, $p = 0.02$ (Forebrain), $F_{2,37} = 5.5$, $p = 0.008$ (Midbrain), $F_{2,37} = 10.4$, $p < 0.001$ (Prepontine), and $F_{2,37} = 6.2$, $p = 0.005$ (Medulla). Letters indicate genotype groups that were significantly different in post-hoc Tukey tests.

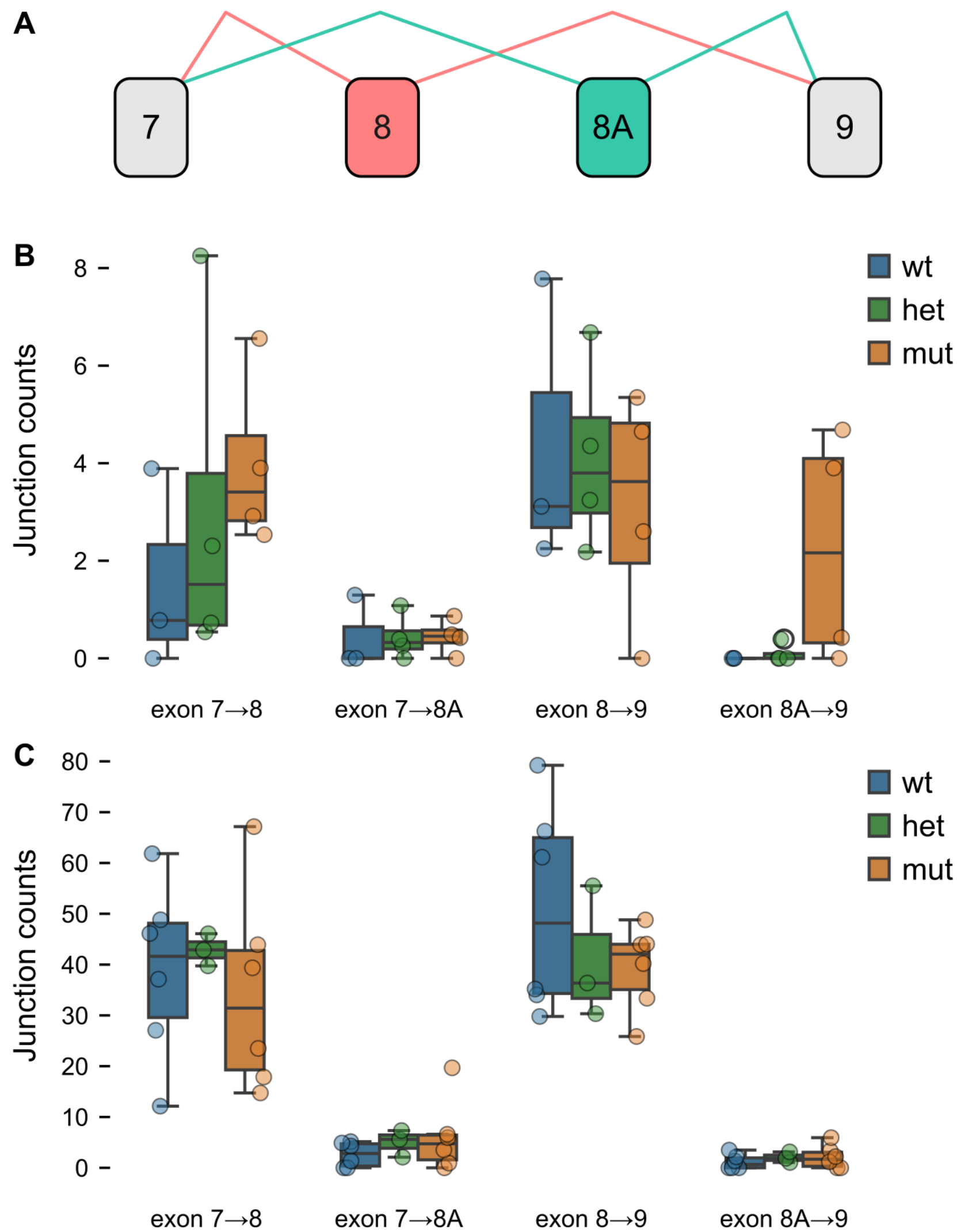

Supplementary Figure 2. Splicing in TS2 zebrafish. (**A**) Alternative splicing of *cacna1c* exons 8 and 8A. (**B-C**). Normalized junction counts from RNA-seq for exon 7 to either exon 8 or 8A, and exon 8 or 8A to exon 9 in wild-type, heterozygous and mutant TS2 larvae, at 2 dpf (B) and 6 dpf (C). Between-group differences not significant for any junction. For box and whisker plots, box shows the 25-75 percentile range, and whiskers show 1.5x the interquartile range.

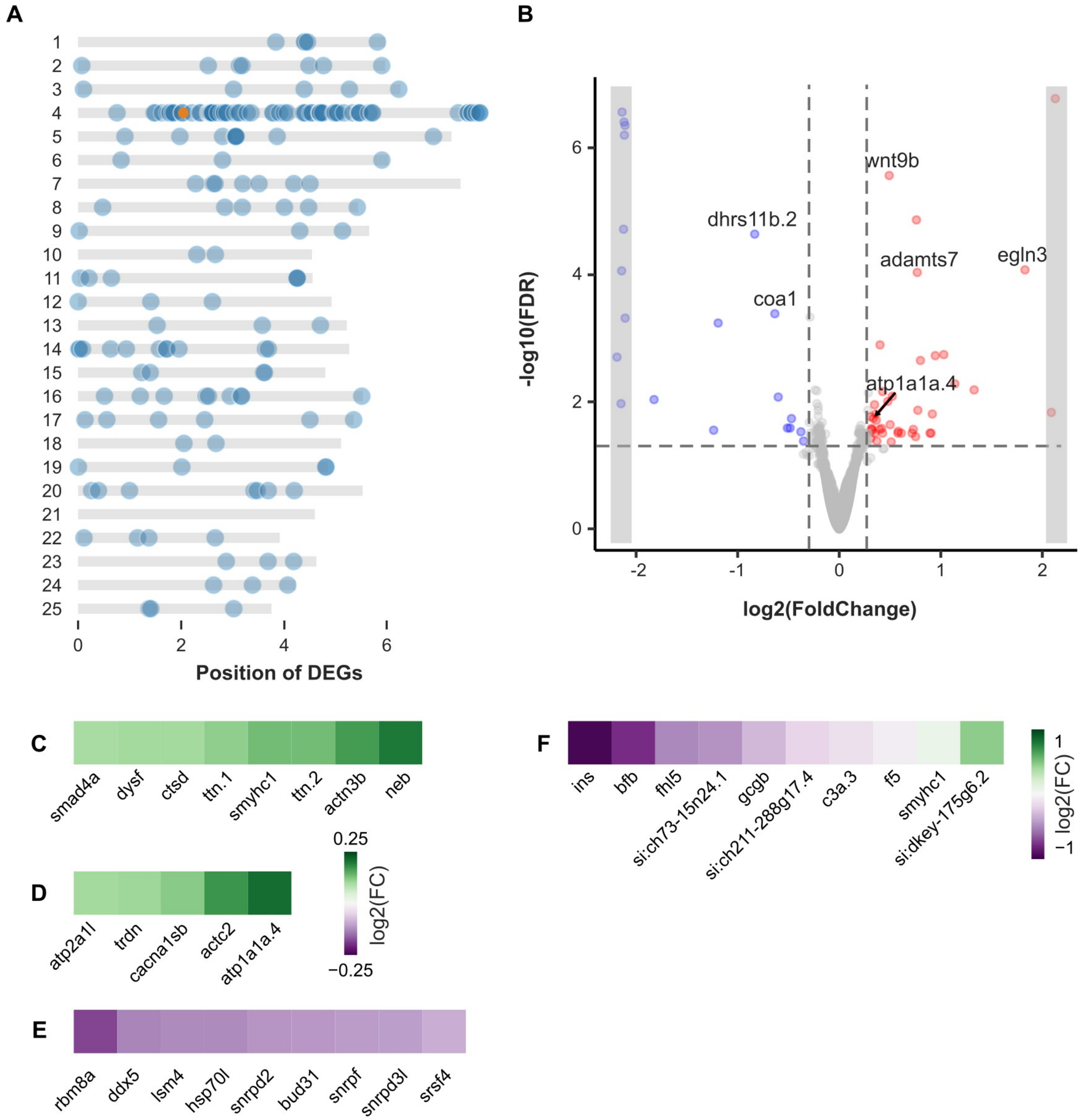

Supplementary Figure 3. Differentially expressed genes in TS2 mutants. (**A**) Chromosomal location of differentially expressed genes in homozygous mutant larvae compared to wild-type siblings (blue markers). Location of *cacna1c* indicated (orange marker). (**B**) Volcano plot for genes differentially expressed between wild-type and homozygous mutants in 2 dpf larvae using RNA-seq of anterior tissue section (head and heart). Values in grey rectangles truncated for display. (**C-E**) Log2 fold change in expression in 2 dpf mutants for genes involved in muscle development (C), cardiac muscle contraction (D) and mRNA splicing (E). (**F**) Change in expression for genes that were significantly different between heterozygous and wild-type sibling 6 dpf larvae.

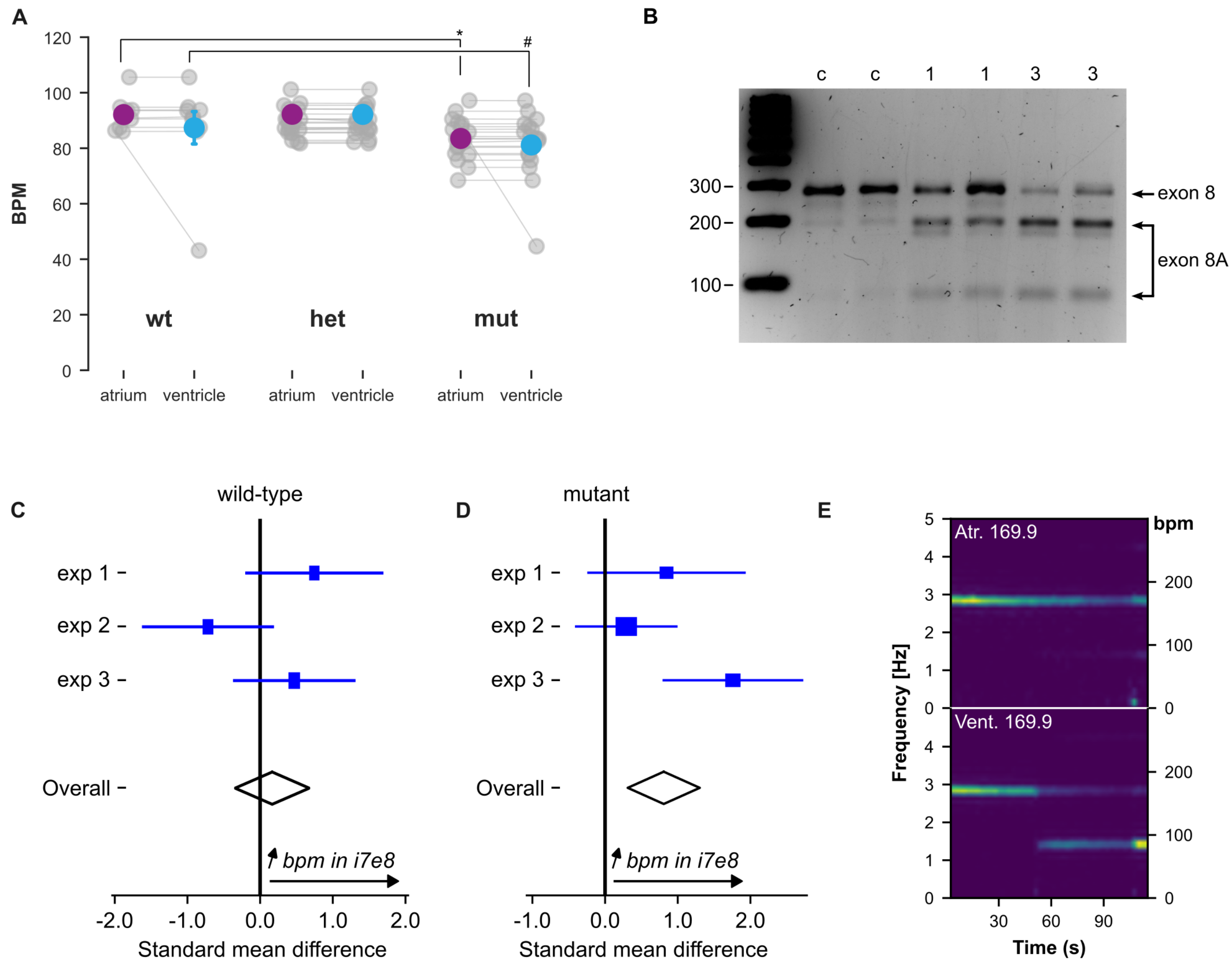

Supplementary Figure 4. Ventricular bradycardia and antisense control of exon 8 splicing. (**A**) Contraction frequency (beats per minute, bpm) in sibling wild-type, heterozygous and homozygous mutant larvae. Atrial frequency was significantly reduced in mutants (Kruskal Wallis H_2 = 11.5, p=0.003, *, Dunn's post-hoc test p = 0.011) but not in heterozygous larvae, and a similar effect was seen for ventricle frequency (Kruskal Wallis H2 = 11.2, p = 0.004. #, Dunn's post-hoc test p = 0.014. Error bars show standard error of mean. (**B**) RT-PCR and restriction digest for 2 dpf embryos injected with control morpholino (c), 1 ng per embryo i7e8 morpholino (1) or 3 ng i7e8 morpholino (3). (**C-D**) Meta-analysis of three experiments injecting morpholinos into wild-type (C) and mutant (D) embryos. Standard mean difference (square) and confidence interval (bars) for atrial contraction frequency between control and i7e8 morphants is shown for each experiment, with diamonds showing overall effect, which was significant in mutants (standard mean difference 0.81, confidence interval [0.31, 1.3]). (**E**) Spectrogram for an individual mutant larva at 6 dpf, where the atrium:ventricle contraction frequency shifted from 1:1 to 2:1 during a two minute recording.

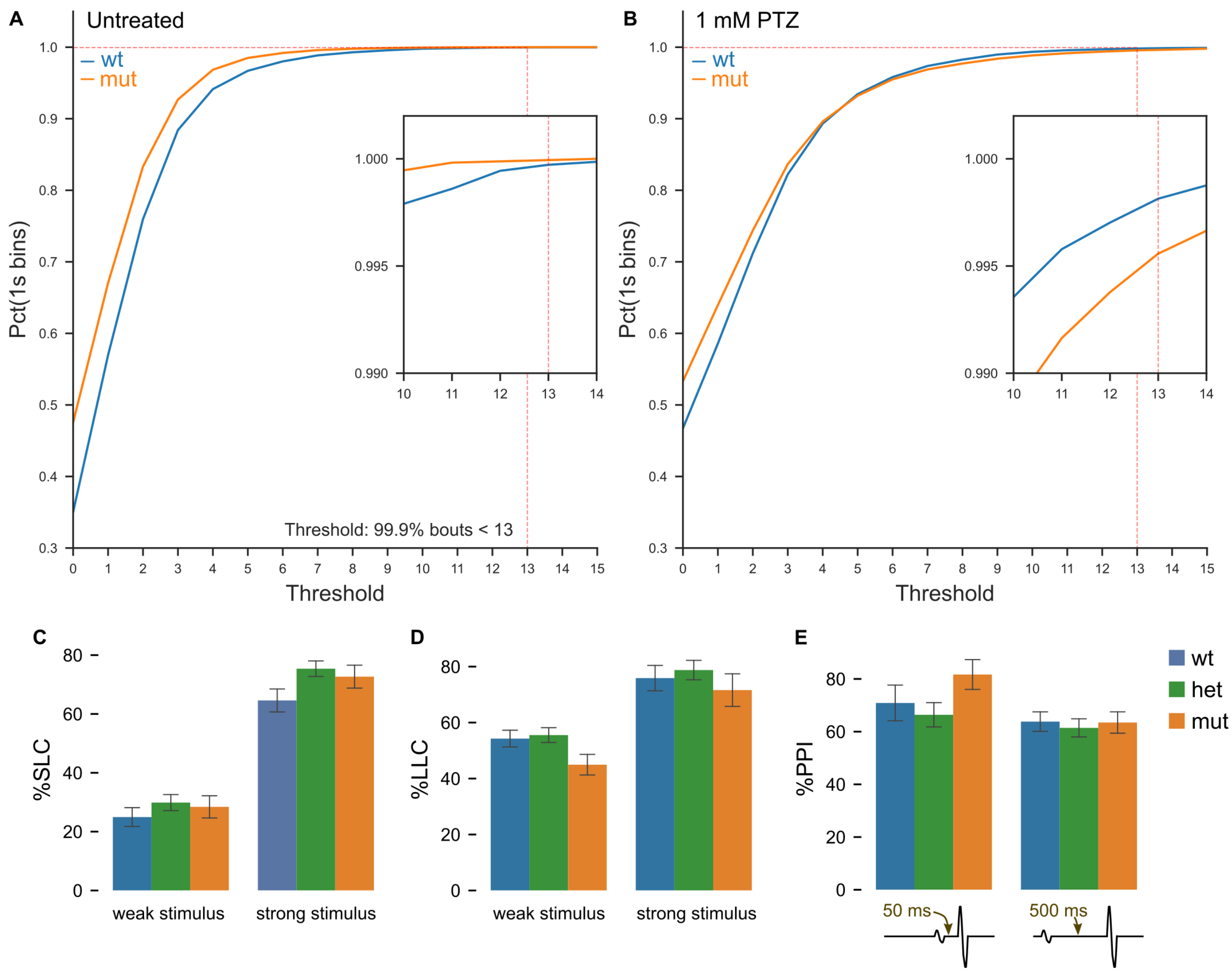

Supplementary Figure 5. Behavioral analysis. Identification of seizure-like activity (A-B). Cumulative histogram of swim distances per 1s recording bin for untreated wildtype sibling larvae (blue, A). Based on this data, we select a threshold exceeded by only 0.1% of windows in wildtypes (dotted red line). Untreated homozygous mutants show a similar low level of large-amplitude events (orange, A). In contrast, in larvae treated with 1 mM PTZ, a larger fraction of swim events exceed this threshold in wildtype larvae, and even more so in mutants (B). Inset figures show same data with different axis limits.(**C-E**) Auditory C-start responsiveness (C-D) and prepulse inhibition (E) in *cacna1c* wild-type, heterozygote and mutant larvae (N= 48, 82 and 37). Larvae perform two distinct types of auditory C-start response distinguished by latency: short latency C-starts (C) and long-latency C-starts (D). No significant effects of mutation were observed for either mode of response, either at low or high intensity auditory stimuli. (**E**) Prepulse inhibition of the startle response at 50 ms (left) and 500 ms (right) interstimulus intervals between prepulse and startle inducing stimulus, tested in the same larvae as above. No differences are significant. Error bars show standard error of mean.

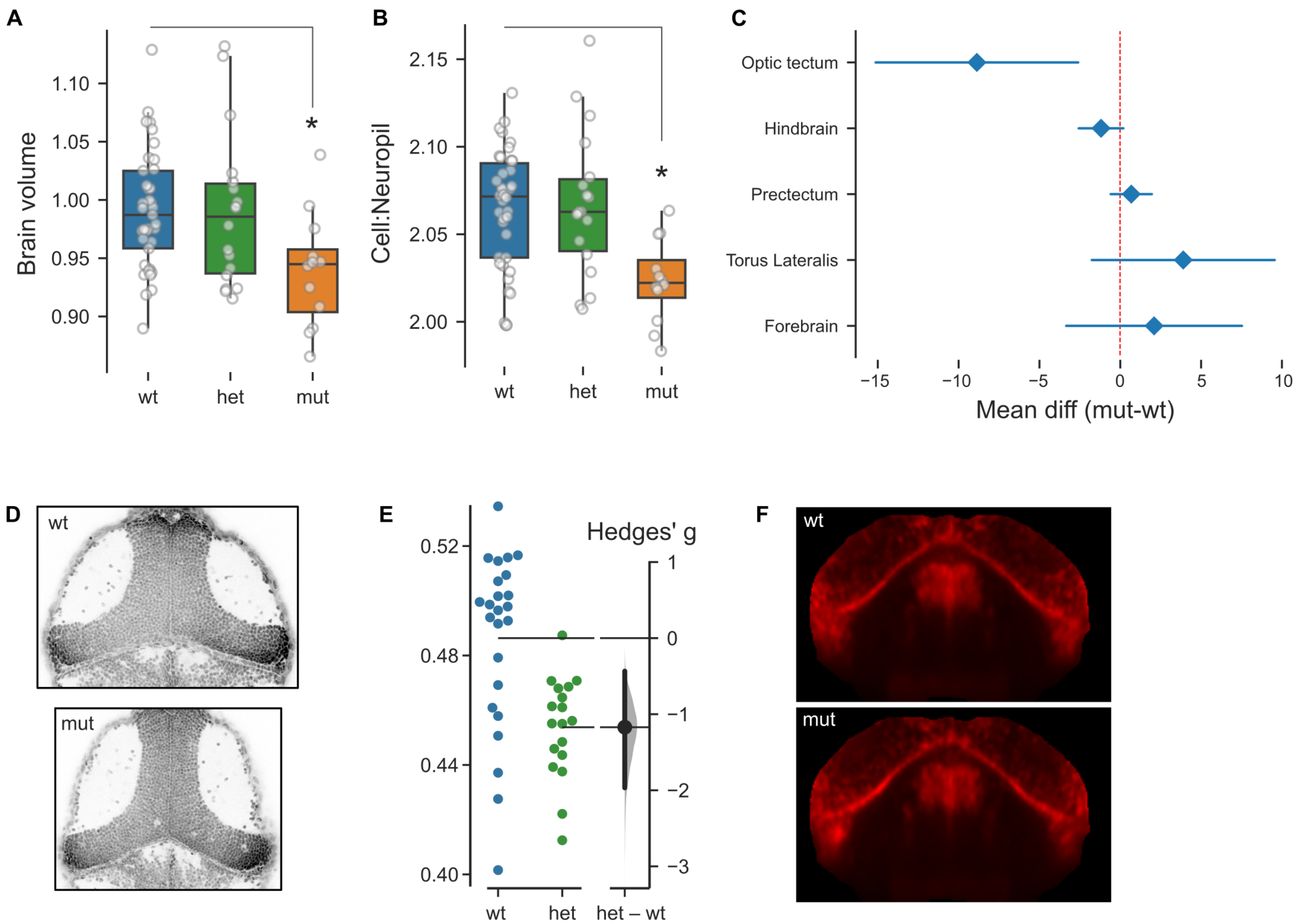

Supplementary Figure 6. Brain volume and cell density. (**A**) Normalized brain volume in wild-type (N=37), heterozygous (N=18) and mutant (N=12) larvae. Each brain volume was normalized to the mean of the wild-type group. Kruskal Wallis H2 = 7.0, p = 0.03. * Dunn's post-hoc test p < 0.01, comparing wild-type and mutants. (**B**) Ratio of the volume of primarily cellular brain areas to neuropil dense areas, same larvae as in (A). ANOVA $F_{2,65} = 7.2$, p = 0.001. * post-hoc Tukey test p = 0.002. For box and whisker plots, box shows the 25-75 percentile range, and whiskers show 1.5x the interquartile range (**C**) Mean (diamond) and confidence interval (bars) for difference in cell density for five brain regions in wild-type and mutant larvae (N=16,14 respectively), after Hoechst33342 staining and manual cell counting. (**D**) Hoechst 33342 stained confocal section through the optic tectum in a wild-type and mutant larva. (**E**) Volume of the midline cerebellar region that was reduced in TS2 homozygous larvae, in an independent cohort of wild-type (N=24) and sibling heterozygous (N=18) larvae. Mask volume normalized to total brain volume. (**F**) Mean signal for *sox2* fluorescent *in situ* hybridization at the same coronal section as in Fig. 6D for wild-type (N=10) and mutant (N=9) TS2 larvae. Horizontal confocal scans were co-registered to ZBB, normalized, averaged and re-sliced for coronal view.

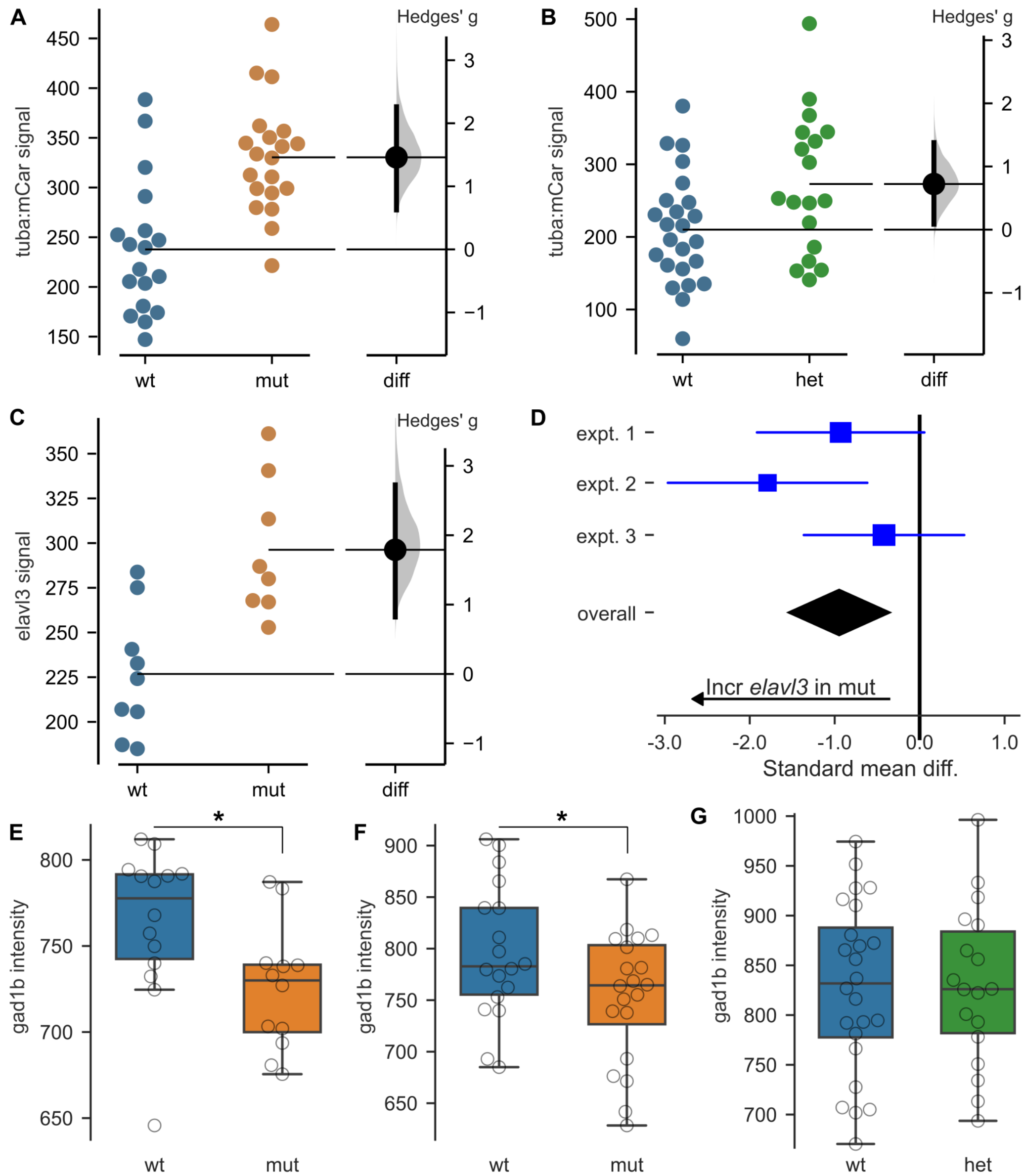

Supplementary Figure 7. Distribution of post-mitotic neurons. (**A-B**) Mean *tuba:mCardinal* signal within a mask encompassing part of the anterior hindbrain midline area with increased expression in Fig. 7A, in an independent cohort of wild-type and homozygous mutant larvae (N=18, 20 respectively ; t-test, $p < 0.001$; A), and in wild-type and heterozygous larvae (N=24,18 respectively ; t-test, $p = 0.023$; B). (**C**) Midline *elavl3* fluorescent *in situ* hybridization signal intensity in the anterior hindbrain midline mask, in wild-type and mutant larvae (N=9 per genotype ; t-test, $p = 0.001$). (**D**). Meta-analysis of three experiments measuring anterior hindbrain midline *elavl3* signal increase in TS2 mutants relative to wild-type larvae. X-axis: standard mean difference (squares) and confidence interval (bars) for signal intensity difference between wild-type and mutant larvae in each experiment. Diamond: overall effect (standard mean difference -0.95, confidence interval [-1.54,-0.36]). Moderate heterogeneity across experiments ($I^2 = 37.2\%$). (**E-G**) Subpallial *gad1b:RFP* fluorescence in wild-type and mutant larvae (E-F, independent cohorts) and wild-type versus heterozygous larvae (G). * Mann Whitney U test (E) and Welch test (F), $p < 0.05$. Box shows the 25-75 percentile range, and whiskers show 1.5x the interquartile range.

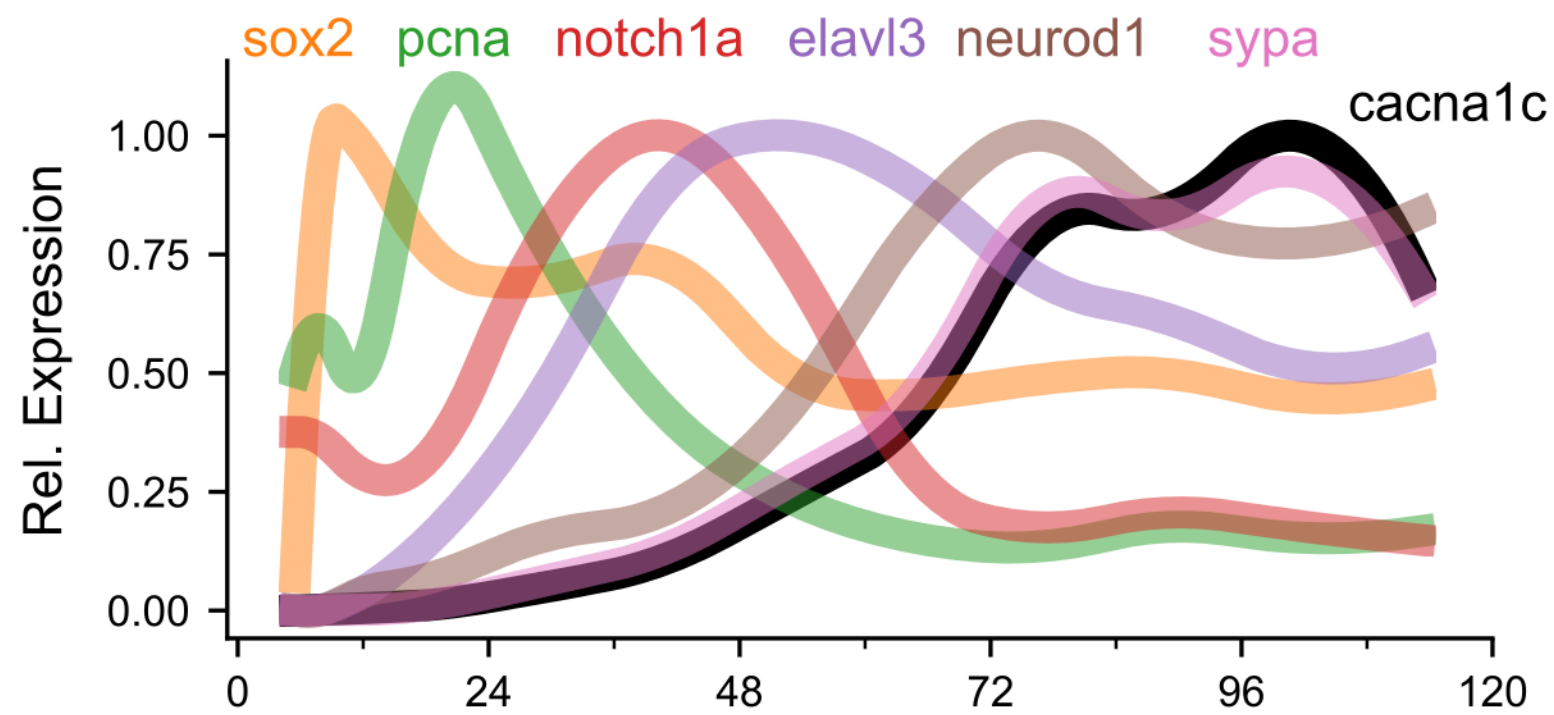

Supplementary Figure 8. *Cacna1c* expression during brain development in zebrafish. Expression of *cacna1c* during embryonic and early larval brain development (0-120 hpf) compared to markers of neuronal maturation. Data is mean read counts per stage, using cells annotated as neural in DanioCell, smoothed and normalized to maximum expression for each gene.

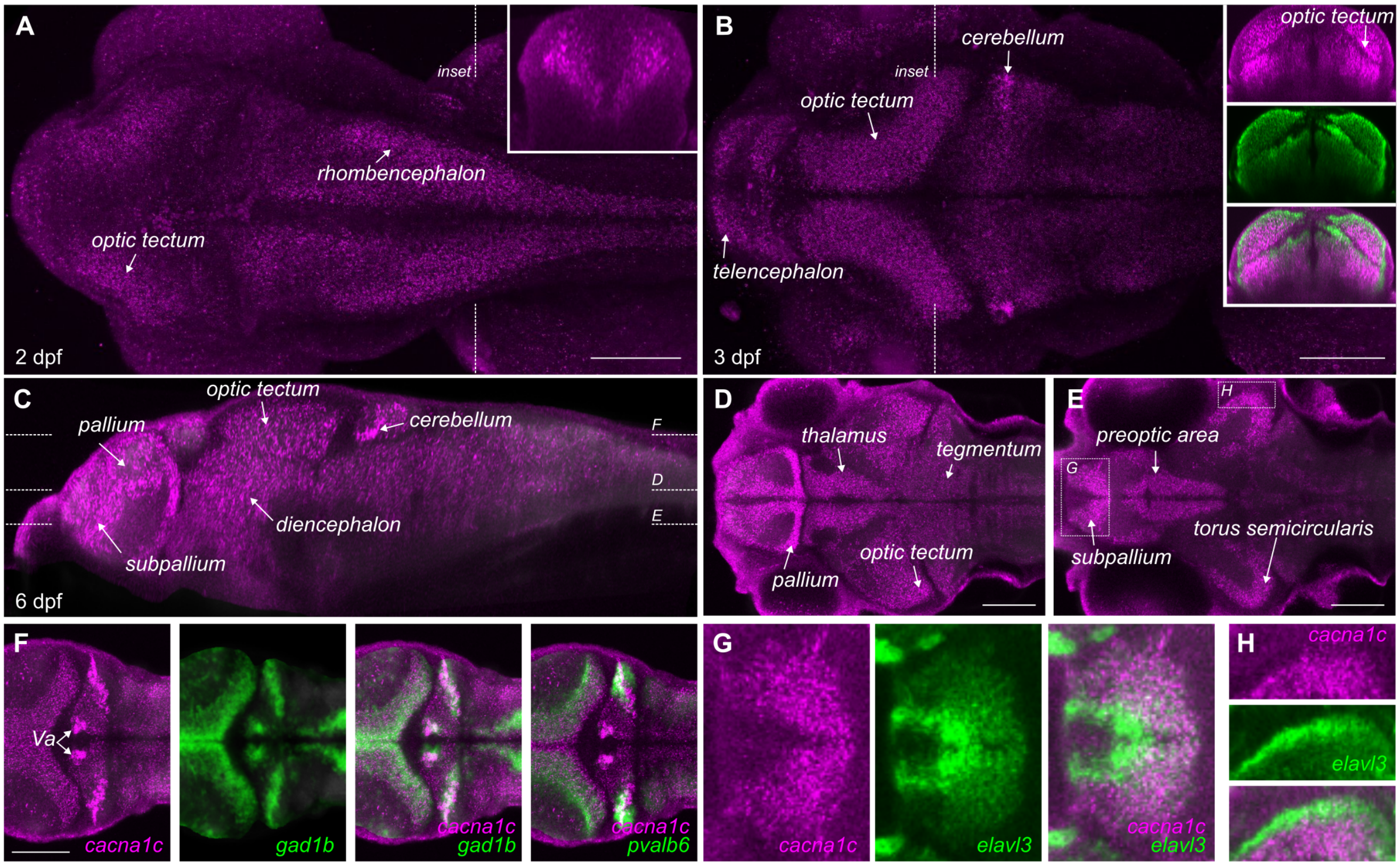

Supplementary Figure 9. Enriched *cacna1c* expression in cerebellar neurons during neural development. (**A-B**) Maximum projection of confocal stacks of *cacna1c* expression from *in situ* HCR at 2 dpf (A) and 3 dpf (B). Insets show coronal re-section at the level indicated by the dotted lines. In (B), inset shows co-*in situ* hybridization for *elavl3* (green). Scale bars 100 µm. (**C**) Sagittal re-section of confocal image stack showing *cacna1c* expression at 6 dpf (mean of 5 co-registered brain images from individual larvae), with dotted lines indicating levels of horizontal slices in (D-F). (**D-E**) Horizontal confocal sections showing *cacna1c* expression in midbrain and forebrain. Scale bar 100 µm. (**F**) Horizontal confocal section through the cerebellum, showing co-registered expression of *cacna1c* (magenta) with *gad1b* and *pvalb6* (green). Scale bar 100 µm. (**G-H**) Horizontal section showing expression of *cacna1c* (magenta) relative to co-labeling with *elavl3* (green) in the subpallium (G) and torus semicircularis (H).

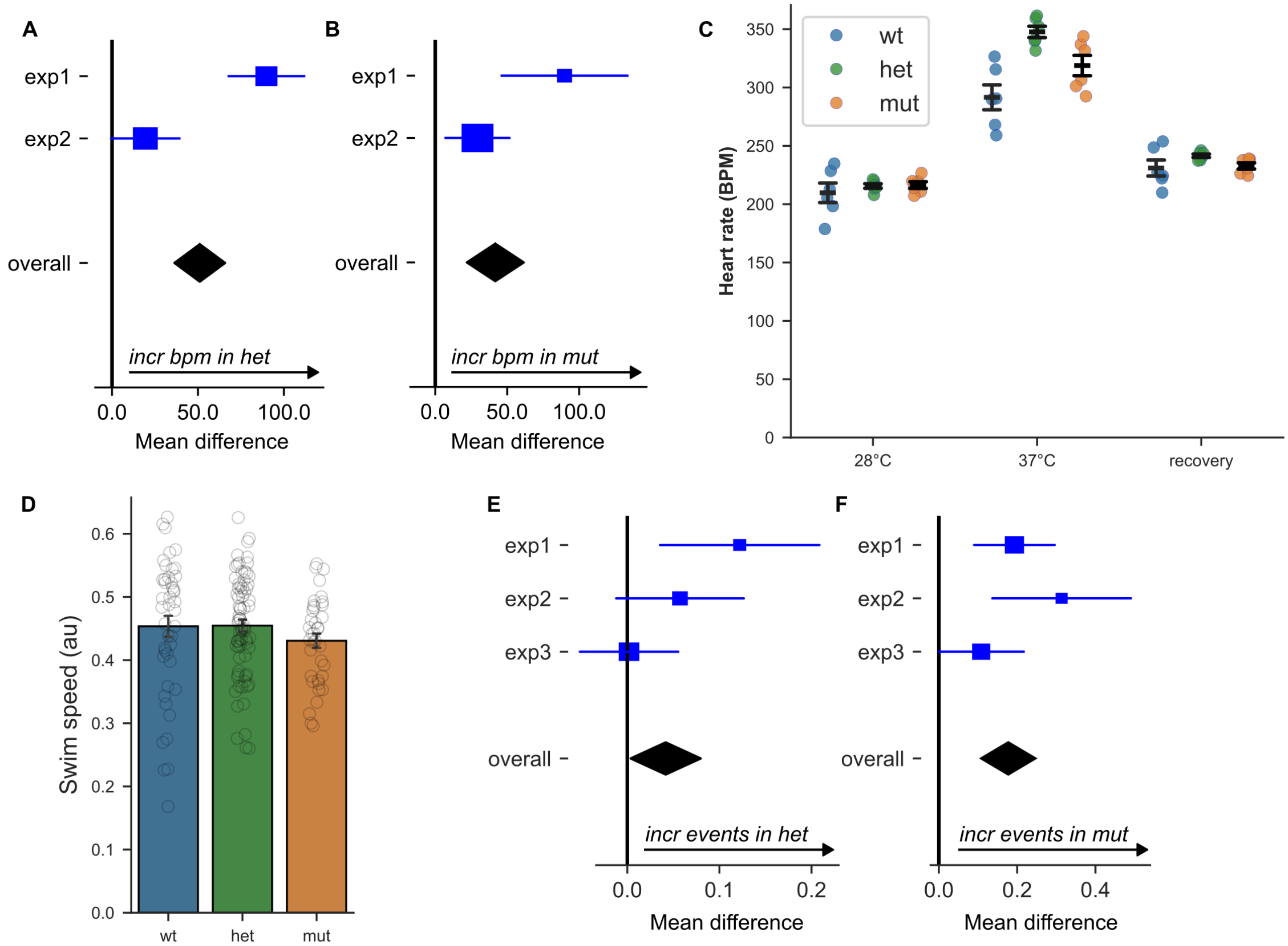

Supplementary Figure 10. Tachycardia and seizure-like activity in heterozygous TS2 larvae at elevated temperature and reversal of tachycardia after return to physiological temperature. (**A-B**) Meta-analysis of two experiments measuring cardiac contraction frequency increase over the wild-type level in TS2 heterozygous (A) or homozygous (B) sibling larvae. X-axis shows mean difference (squares) and confidence interval (bars) for difference between groups in each experiment. Diamond shows significant combined effect. A: mean difference 51 bpm, confidence interval [36.4,65.8], $p < 0.001$; B: mean difference 41.7 bpm, confidence interval [22.0,61.4], $p < 0.001$. (**C**) Heart rate in wild-type larvae and heterozygous and homozygous mutant siblings (N = 6 larvae for each genotype) at 28°C, 37°C, and 28°C 90 min after heat exposure ("recovery"). (**D**) Mean swim speed during active swimming for larvae at 37°C for wildtype (N=44), heterozygous (N=73) and mutant (N=37) larvae. (**E-F**) Meta-analysis of three experiments measuring seizure-like behavior: increase over the wild-type level for heterozygous (E) or mutant (F) sibling larvae. E: mean difference 0.04 events per minute, confidence interval [0,0.08], $p = 0.032$. F: mean difference 0.18, confidence interval [0.11, 0.25], $p < 0.001$.

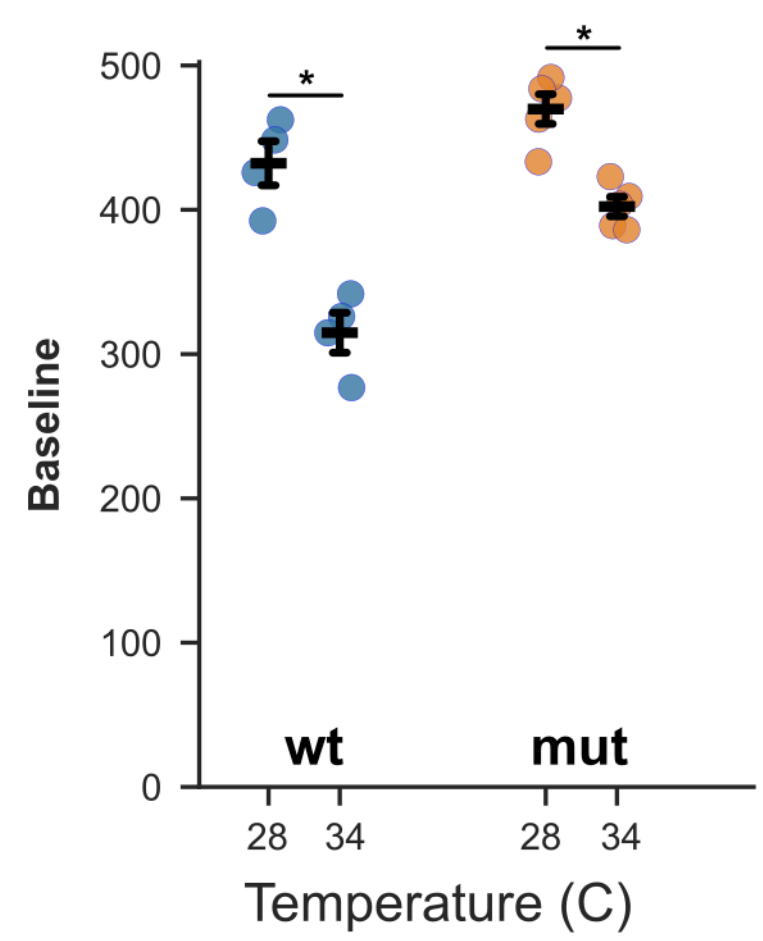

Supplementary Figure 11. Baseline GCaMP fluorescence intensity decreases at elevated temperature. Baseline GCaMP fluorescence (arbitrary units) in wild-type and homozygous mutant larvae at the indicated temperatures. N = 4,5 for wt and mutant animals. Repeated measures ANOVA, main effect of temperature $F_{1,7} = 353$, $p < 0.001$. Post-hoc comparisons * $p < 0.001$.

Supplementary Table 1. qPCR primers for measuring exon 8 and 8A usage in *cacna1c*

Target	Forward primer	Reverse primer	Product size
ef1α (reference)	CTGGAGGCCAGCTCAAACAT	ATCAAGAAGAGTAGTACCGCTAGCATTAC	87
cacna1c exon 8	ATCTGGTTCTGGGTGTGTTG	TCGTCGTCATTCTCAGGGTC	169
cacna1c exon 8A	TGAATGATGCTGTAGGGAATTCC	TTAGATCCTCCTCCAGCTGC	189
