# Supplementary material for: Elevated body temperature exacerbates arrhythmia and seizure-like activity in a zebrafish model of Timothy syndrome
Source: Commun Biol. 2026 Jul 9;9:1442. doi: 10.1038/s42003-026-10657-3 (PMC13724642; doi:10.1038/s42003-026-10657-3)
Supplement: Supplementary file 3 [file 42003_2026_10657_MOESM2_ESM.pdf]

Description of Additional Supplementary files

File name: Supplementary Data 1.

Description: Differential gene expression analysis at 2 dpf comparing wild-type and homozygous mutant larvae. Comparison of all genes except for those on chromosome 4.

File name: Supplementary Data 2.

Description: Differential gene expression analysis at 6 dpf. First sheet shows comparison for wildtype and homozygous mutant larvae. Second sheet shows comparison for wild-type and heterozygous larvae. Comparison of all genes except for those on chromosome 4.

File name: Supplementary Data 3.

Description: Oligonucleotide sequences for probes used to perform HCR fluorescent in situ hybridization.

File name: Supplementary Data 4.

Description: Numerical data underlying graphs and charts.

File name: Supplementary Video 1

Description: Elevated brain activity in mutants. Fluctuating GCaMP fluorescence signal in example wild-type (top) and homozygous mutant (bottom) larvae recorded over 5 min.

File name: Supplementary Videos 2-3

Description: Atrium and ventricular contractions. Videos showing atrial (blue outline) and ventricular (orange outline) contractions in wild-type (S2) and homozygous mutant (S3) larvae, slowed to half speed.
